# Emerging Role of Flavonoids as the Treatment of Depression

**DOI:** 10.3390/biom11121825

**Published:** 2021-12-03

**Authors:** Arzoo Pannu, Prabodh Chander Sharma, Vijay Kumar Thakur, Ramesh K. Goyal

**Affiliations:** 1 Department of Pharmacology and Toxicology, School of Pharmaceutical Sciences, Delhi Pharmaceutical Sciences and Research University (DPSRU), New Delhi 110017, India; arzoopannu@gmail.com; 2 Department of Pharmaceutical Chemistry, School of Pharmaceutical Sciences, Delhi Pharmaceutical Sciences and Research University (DPSRU), New Delhi 110017, India; sharma.prabodh@gmail.com; 3 Biorefining and Advanced Materials Research Centre, Edinburgh EH9 3JG, UK; 4 School of Engineering, University of Petroleum & Energy Studies (UPES), Dehradun 248007, India

**Keywords:** nutraceuticals, depression, flavonoids, anti-depressants, anti-oxidant

## Abstract

Depression is one of the most frequently observed psychological disorders, affecting thoughts, feelings, behavior and a sense of well-being in person. As per the WHO, it is projected to be the primitive cause of various other diseases by 2030. Clinically, depression is treated by various types of synthetic medicines that have several limitations such as side-effects, slow-onset action, poor remission and response rates due to complicated pathophysiology involved with depression. Further, clinically, patients cannot be given the treatment unless it affects adversely the job or family. In addition, synthetic drugs are usually single targeted drugs. Unlike synthetic medicaments, there are many plants that have flavonoids and producing action on multiple molecular targets and exhibit anti-depressant action by affecting multiple neuronal transmissions or pathways such as noradrenergic, serotonergic, GABAnergic and dopaminergic; inhibition of monoamine oxidase and tropomyosin receptor kinase B; simultaneous increase in nerve growth and brain-derived neurotrophic factors. Such herbal drugs with flavonoids are likely to be useful in patients with sub-clinical depression. This review is an attempt to analyze pre-clinical studies, structural activity relationship and characteristics of reported isolated flavonoids, which may be considered for clinical trials for the development of therapeutically useful antidepressant.

## 1. Introduction

Among mental disorders, depression is one of the most commonly known multifaceted disorders which negatively impacts social life, work and health of humans [1,2]. It is the most responsible cause of suicide or illness occurring across all ages, despite of social background and gender [3]. A variety of antidepressants are available for treating depression such as tricyclic antidepressants, selective dopamine reuptake inhibitors, selective norepinephrine reuptake inhibitors and SSRIs (selective serotonin reuptake inhibitors) [4,5]. They exert their anti-depressant action via acting on various neurotransmitter systems, including serotonergic (5-HT), dopaminergic (DA), and noradrenergic (NA) or by inhibiting mono amino oxidase (MAO) enzymes [6]. These antidepressants may, however, have delayed onset of action and possess multiple side effects when given for a longer duration [7]. Further, some of these are found to be less effective in the treatment-resistant depression and in some cases, there is incomplete recovery of patient with these standard medicines [8].

In case of sub-clinical depression, during the initial stage of development of major depression, it is difficult to treat the person clinically with synthetic anti-depressants [9]. Even, in several cases, ‘sub-clinical depression’ arises as a clinical condition of patients who have not yet entered full remission and has symptoms left over after treatment with synthetic antidepressants [10,11]. In addition, symptoms of sub-clinical depression are also very common in general people, among those who do not have any clinically diagnosed mental illnesses [12]. Due to lacking in diagnostic parameters for subclinical depression, the associated symptoms affect quality of common life, social life and occupational life functioning, resulting in economic and personal encumber [13]. Therefore, there is a need for consideration or provision for new basic or coordinated approaches to diagnose sub-clinical depression with investigation of specific interventions or treatment options for prevention of sub-clinical depression. Several emerging pieces of evidence have shown an association between dietary measures and the risk of depression and also, have suggested that adding nutrients in diet can affect the onset of depression [14,15]. Even many clinical trials have lightened up the effectiveness of nutraceuticals as antidepressants in the initial phase of depression or sub-clinical depression, via providing a wide range of pharmacological effects [15,16,17].

By identifying these important neurobiological mechanisms of nutraceuticals, this adjunctive therapy can be proven scientifically to have potential antidepressants effect. There are different mechanisms suggested to be involved in neuroprotective effect of nutraceuticals (i.e., fats, amino acids, minerals, vitamins and other nutrients) via playing a remarkable role in nurturing structure and function of neurons, provide energy and nutrients to brain, strengthen the immunity, exert antioxidant defence mechanism, influence neurotrophic factor essential for maintaining brain plasticity and neuronal preservation [18,19,20]. Nutraceuticals are found to modulate neurobiological pathways involved in depression, such as monoamine deficiency, reduced neurogenesis, bioenergetics abnormalities; cytokine alteration associated with chronic inflammation neuro-endocrinological disorders [21,22]. Therefore, scientific evidence seems to regard food as a supplement and as a key to the approach to mental illness.

Admonition related to the nutraceuticals prescription includes the significance of prescribing highly standardized, stable and high-quality nutrient products with appropriate formulas and doses. The practitioners may be confused about which products to be prescribed or recommended, and in such type of cases it is advisable to refer specialized health professionals having the knowledge of nutraceuticals. However, it is reasonable or comprehensible to stand by to gauge the dose, potency and mechanism of anti-depressants before the introduction of prescription nutraceuticals to any patient, so that nutraceuticals could be started as the first antidepressant treatment [23,24,25,26]. All these factors have prompted researchers to find plant base alternatives for antidepressant action. Therefore, in this review, we have tried to emphasize the potential anti-depressant outcome of precious flavonoids isolated from plants or herbs.

Flavonoids are natural occurring polyphenols that have been extensively investigated for their pharmacological properties [27]. Almost all fruits, grains, vegetables, alcohol and tea are the rich source of flavonoids and are capable of preventing or reversing stress through a number of mechanisms [28]. In past decades, a large number of studies have conducted to explore antidepressant activity of natural chemical compounds, especially flavonoids, having multiple actions on brain [29]. Several preclinical studies have shown that certain flavonoids have antidepressant potential and have found to reverse depressive behavior of rodents in animal models. Increasing in expression levels of various neurotransmitters, neurotrophic factors and neurogenesis in brain are the suggested underlying mechanisms for anti-depressant action [1,30]. In the present review, we have focused antidepressant potential of certain flavonoids and to describe the mode of action involved based on pre-clinical studies. The aim of the present review chapter is to compile potential molecules from literature having beneficial antidepressant action, which would help to develop effective and safe nutraceuticals products to reduce depression in humans.

## 2. Flavonoids and Structure Activity Relationship

**Figure 1:**
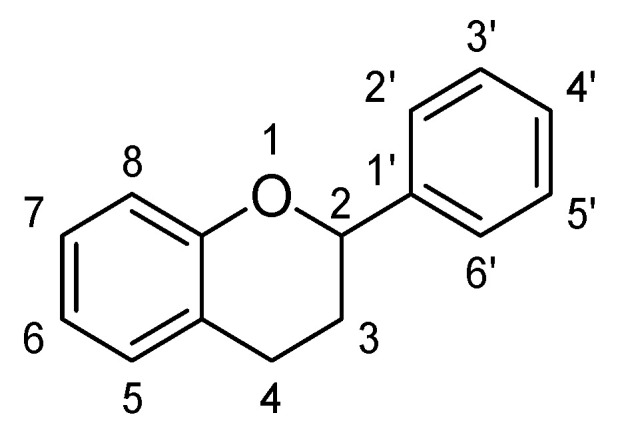
Skeleton of Flavonoids.

Flavonoids are low molecular weight phenolic compounds and widely found in plants. To date, more than 5000 flavonoids are identified, which have extensive biological activity [1]. On the basis of their respective structures, flavonoids could be categorized into major groups, i.e., flavones, flavonols, isoflavones, flavanones, flavanonols, flavanols and anthocyanidins [31]. Generally, a flavonoid skeleton contains two aromatic carbon rings, one benzene and benzopyran, as shown below. This classification is related to hydroxylation manner of the ring structure, oxidation level of the C-ring and substitution at 3-position (Figure 1). Therefore, variation in the number and sequence of hydroxyl groups, with their glycosylation and alkylation is the responsible factor for the differences among these classes [32]. Various pre-clinical and in-vitro trials have shown several pharmacological aspects of flavonoids, i.e., they are antioxidant, hepatoprotective, antiallergic, anticarcinogenic, antiviral, neuroprotective, antitoxic, antiepileptic, anti-angiogenic, anti-diabetic and estrogenic [33,34,35,36,37,38,39]. These actions depend upon type and dose of flavonoids administered [1].

### 2.1. Flavones

**Figure 2:**
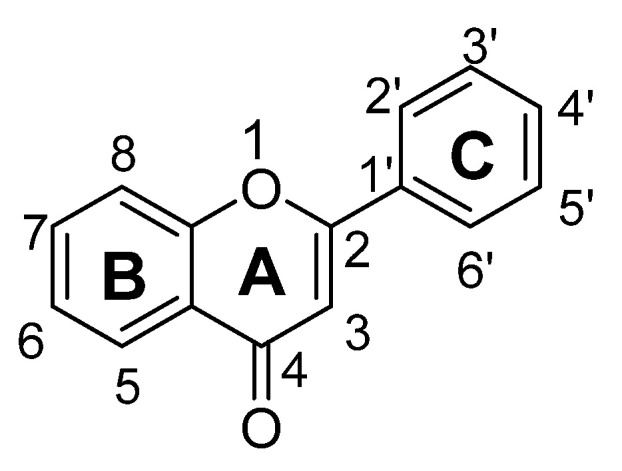
Skeleton of Flavones.

Flavones are one of the most interesting flavonoids because of their in-vivo and in-vitro biological activities. The basic backbone of flavones is 2-phenylchromen-4-one (2-phenyl-1-benzopyran-4-one) (Figure 2). Unlike other flavonoids, flavones have no substitution at position C3, and having double bond between positions C2 and C3. In addition, they oxidized at position C4. Usually, flavones predominantly present in plants as glycosides composed of aglycone plus sugar moiety [40]. These can be easily hydrolyzed with acid or enzyme such as malonyl esterases and remains as glycoside after heating, shredding or juicing. Whereas unlike flavone O-glycoside, flavone C-glycoside are more stable during process such fermentation, juicing, heating, pasteurization and spray drying. Therefore, all these parameters should be considered during isolation of flavones 7-O-, 6-C- and 8-C-glucosides [37,38,39,40].

Various flavones have been isolated from different plants and herbs to evaluate anti-depressant potential using different animal model and found to be significant candidate to combat depressive-like behavior in rodents (Table 1). These flavones act as anti-depressant via different mechanism and have not shown any adverse effect during studies.

**Figure 3:**
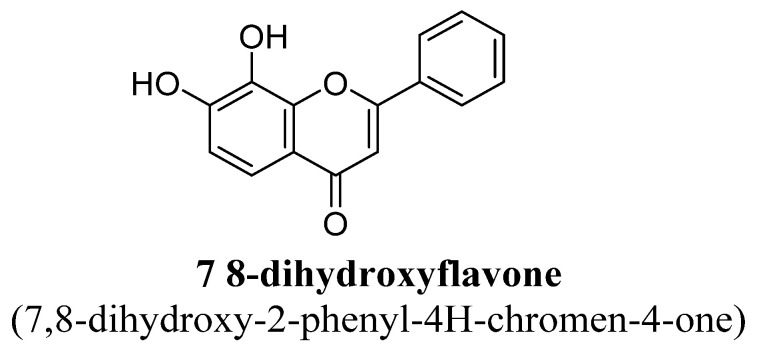
7, 8-dihydroxyflavone.

Further, 7, 8-dihydroxyflavone (Figure 3) is a natural flavone which has shown antidepressant action in several studies. Pre-clinically, 7, 8-dihydroxyflavone has been reported to mimic BDNF (brain derived neurotrophic factor) and also increased its expression and level in hippocampus [41]. In addition, it has been reported to have therapeutic potential against several neurological diseases or disorders, i.e., stroke, depression and Parkinson’s disease using animal models. In two different studies, 7, 8-dihydroxyflavone has shown significant oral bioavailability and found to cross the brain–blood-barrier (BBB). Hence, this flavone found to be a superior phytoconstituent for treating depression disorder as it acts on nitric oxide signalling pathway and activated TrkB receptors (tropomyosin receptor kinase B), also [42,43].

**Figure 4:**
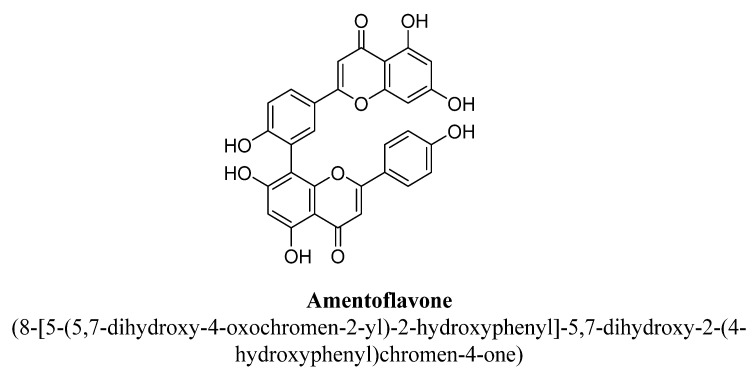
Amentoflavone.

Amentoflavone (Figure 4) is a natural biflavonoid formed by coupling of two molecules of apigenin at position C8 and it have been reported to possess many pharmacological potentials such as neuroprotective action, antioxidative and anti-inflammatory effect [44]. Ishola et al., studied that amentoflavone flavone isolated from methanolic extract of roots of *Cnestis ferruginea* Vahl ex DC. has shown anxiolytic and antidepressant effects in mice. It has been concluded that the underlying mechanism of amentoflavone was via interactions with the ionotropic GABA (gamma-aminobutyric acid), adrenergic receptors (α1- and α2-) and serotonin (5-HT2) receptors [45].

**Figure 5:**
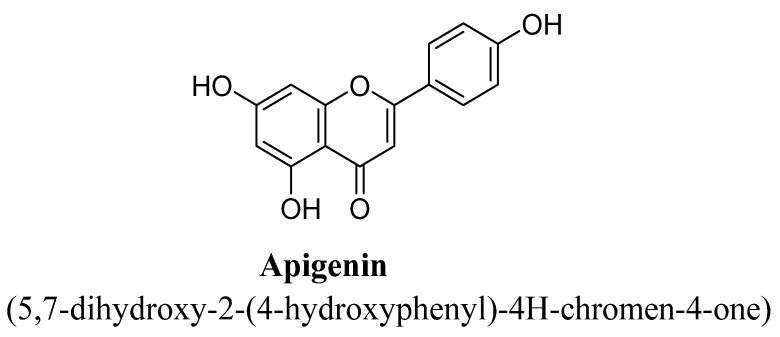
Apigenin.

Apigenin is a 4′, 5, 7-trihydroxyflavone (Figure 5), founds in vegetables and fruits. It has been reported to have several pharmacological actions such as antitumor, anti-inflammatory and antioxidant activities [46]. Several pre-clinical studies were demonstrated to prove anti-depressant potential of apigenin. Nakazawa et al., have studied antidepressant action of apigenin using forced swim test in rodents and found to ameliorate depressive-like behavior in mice, which was suggested to be mediated via dopaminergic system [47]. In another study, apigenin has been found to exhibit antidepressant effects in rats evaluating using chronic unpredictable mild stress animal model. The authors concluded that this effect possibly due to upregulation of PPAR (peroxisome proliferator-activated receptor) expression resulting in inhibition of NLRP3 inflammasome expression and IL-1 production [48]. Involvement of apigenin in up-regulating the level of hippocampal BDNF [49] and inhibition of mono-amino oxidase enzyme has also confirmed its anti-depressant mechanism [50]. Apigenin has also found to reverse the lipopolysaccharide-induced depression in rodents which may be due to its anti-inflammatory potential [51].

**Figure 6:**
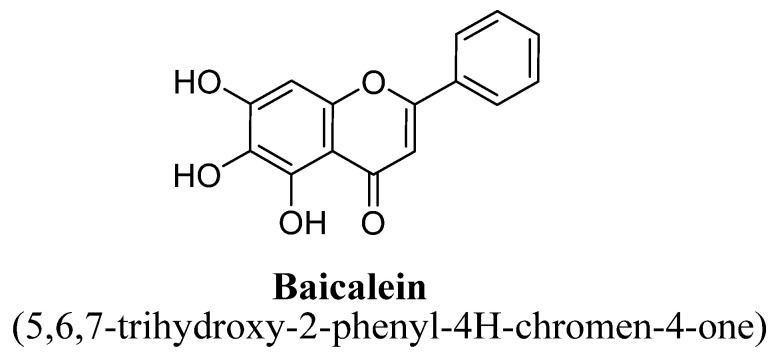
Baicalein.

Baicalein is a 5, 6, 7-trihydroxyflavone flavonoid having hydroxyl group at position C5, C6 and C7 (Figure 6). It is found to be one of the most active flavones among all flavonoids present in *Scutellaria baicalensis* Georgi, [52]. Various reports have proved that baicalein has strong antioxidant activity with significant xanthine oxidase inhibition and free-radical scavenging properties [52,53]. In literature, this flavone has reported to exhibit significant antidepressant effect via reversing the level of reduced ERK phosphorylation and hippocampal BDNF expression in animal model of chronic mild stress [54]. In addition, it has been found to cross the BBB. Another study has also proved the antidepressant potential of baicalein mediating via prevention of decrease level of BDNF and dopamine in hippocampus [55]. Further studies have suggested that baicalein isolated from methanolic extract of roots of *Scutellaria baicalensis* Georgi could inhibit cyclooxygenase-2 in rodent brain resulting in reduced brain level of prostaglandin E2 and act as strong anti-oxidant, which also helps in prevention of the chronic mild stress-induced depression-like behavior in mice [56].

**Figure 7:**
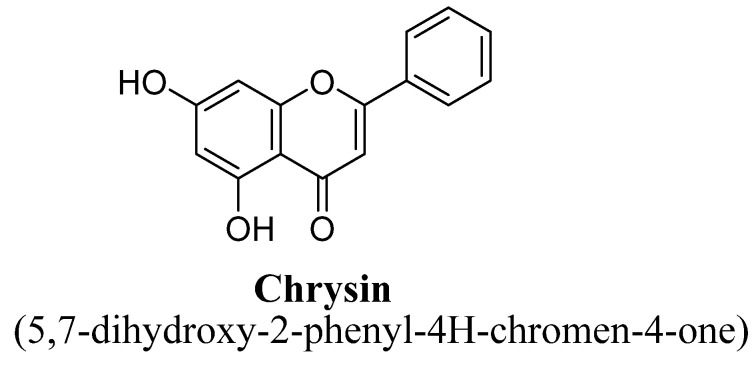
Chrysin.

Chrysin (Figure 7) is a 5, 7-dihydroxyflavone natural flavonoid highly found in honey and many plants, exhibiting multiple pharmacological activities i.e., antineoplastic, anti-inflammatory, antioxidant and hypolipidemic [57,58,59]. In literature, several researchers have demonstrated antidepressant action of chrysin in rodents using chronic unpredictable- mild stress. The proposed underlying mechanism associated with anti-depressant potential of chrysin is up-regulation of BDNF expression and its level in the hippocampus and prefrontal cortex of stressed mice [60]. In another study, researchers have proved anti-depressant potential of chrysin in olfactory-bulbectomized mice and suggested that BDNF was the key target of chrysin in preventing the depressant [61]. Further, the authors suggested chrysin also modulated the 5-hydroxy-tryptamine metabolism, pro-inflammatory cytokines synthesis, caspases activities and kynurenine pathway [62].

**Figure 8:**
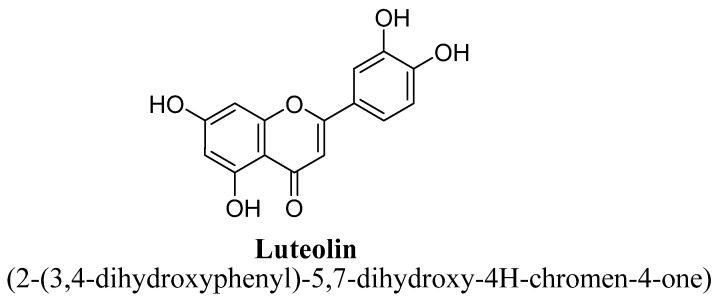
Luteolin.

Luteolin (Figure 8) is a 3′, 4′ 5, 7, tetrahydroxyflavone natural flavonoid and possess various pharmacological properties such as anticancer, antioxidant, anxiolytic, memory-boosting properties, and also found to easily penetrate through BBB [63]. De la Peña et al., have reported that luteolin isolated from ethanolic extract of dried aerial parts of *Cirsium japonicum* Var. Maackii have shown antidepressant effects possibly mediated via potentiation of GABA-A receptor-calcium ion channels [64]. Ishisaka et al., also have confirmed anti-depressant potential of luteolin mediated via attenuation of hippocampal expression of stress-related protein of endoplasmic reticulum using animal model of corticosterone-induced depression [63]. It has been reported to inhibit MAO enzyme, thereby directly leading to increases in neurotransmitter levels in the brain in depression [50].

**Figure 9:**
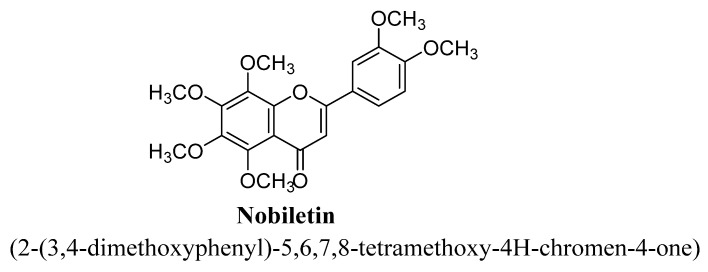
Nobiletin.

Nobiletin is a citrus flavones and chemically known as 5,6,7,8,3′,4′-hexamethoxyflavone (Figure 9). This flavone has been reported to exert neuroprotective effects against β-amyloid peptide-induced neuronal death in hippocampal CA1 region, cognition impairment and also reduced level of β-amyloid peptides [65]. The anti-depressant potential of nobiletin isolated from orange peel was studied in animal models, i.e., forced swim and tail suspension test; and found to significantly inhibit depressive-like behaviors. This anti-depressant action of nobiletin was suggested to be umpired via interaction with noradrenergic (α1-adrenoceptor), dopaminergic (D1 and D2- receptors) and serotonergic (5-HT1A and 5-HT2- receptors) systems [66].

**Figure 10:**
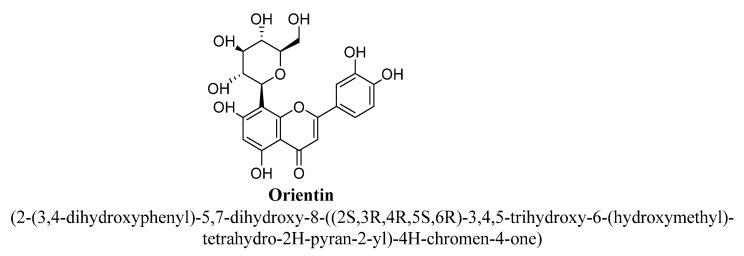
Orientin.

Orientin is a C-glycosyl flavonoid, i.e., luteolin substituted with β-D-glucopyranosyl moiety at C8 position (Figure 10). It is highly abundant in herbs, fruits and millet and found to possess strong antioxidant activity [67]. In a study, anti-depressant effect of orientin was evaluated using animal model of chronic unpredictable mild stress and found to exert antidepressant-like activity in mice. The suggested possible mechanism was increase in MAO inhibition, neurotransmitters level, synaptic proteins and BDNF expression in prefrontal cortex and hippocampus. Further, it has been found to improve neuroplasticity, neurotransmission and reduce oxidative stress in depressed mice [68].

**Figure 11:**
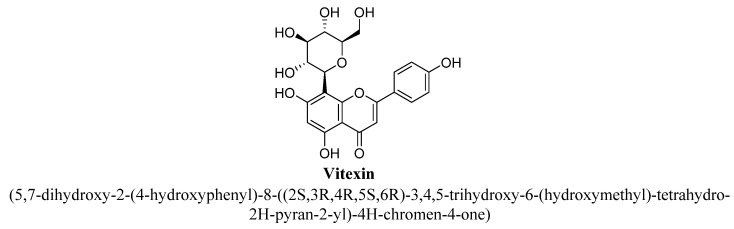
Vitexin.

Vitexin (C-glycosyl compound and a trihydroxyflavone; Figure 11) is an apigenin flavone glucoside and present in nutraceuticals and foodstuffs. In literature, it has been documented to exert many pharmacological actions such as antitumor, anti-inflammatory, peripheral analgesic and antioxidant activities [69,70,71,72]. In addition, it has been found to inhibit platelet aggregation, urease, α-glucosidase and adipogenesis [73,74,75,76]. Among plants, Vitexin is highly abundant in *Passiflora incarnate* L. and reported to have significant anxiolytic and anti-depressant activities mediated via interaction with dopaminergic (D1, D2, and D3), serotonergic (5-HT1A) and noradrenergic (α2) receptors and also by increasing synaptic concentration of neurotransmitters [77].

#### Structure Activity Relationship (SAR) of Flavones

**Figure 12:**
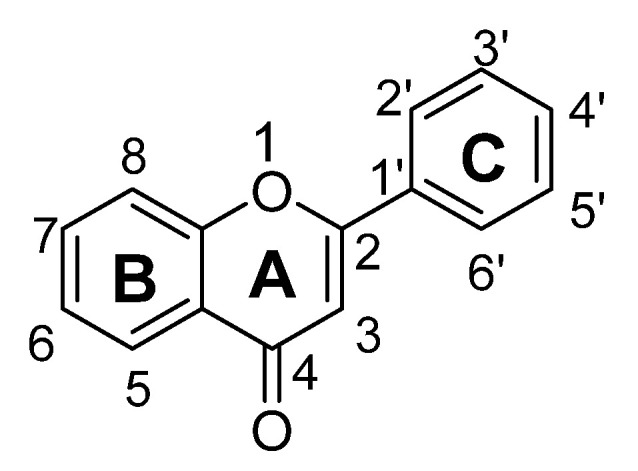
SAR of Flavones.

Based on the information available on the activities of various flavones, following SAR appears to be applicable (Figure 12).

1. The presence of ketonic group at C4 and ring B, C may be responsible for enhancement of BDNF level in the brains of mice.
2. Attachment of hydroxyl group at C7 and long chain group at C8 resulting in increment of activity of serotonin and nor-epinephrine pathways.
3. Hydroxyl group at C3′ and C4′ is necessary for increments of anti-oxidant potential and radical scavenging properties.
4. At C3′ and C4′ position, mono-substitution enhances the selectivity towards MAO-A inhibition, whereas di-substitution increases the selectivity for MAO-B inhibition.
5. Glycoside-O linkage at C7 abolished or reduces the MAO inhibitory potential.
6. Acetate and methyl group at C7 and C8 decreased antioxidant potential of flavones.

### 2.2. Flavonols

**Figure 13:**
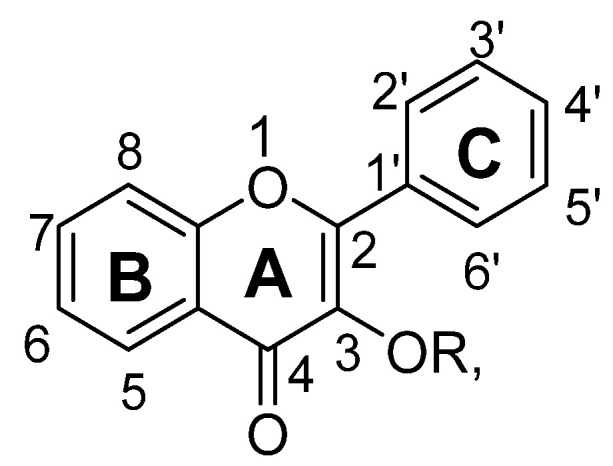
Skeleton of Flavonols.

Flavonols are polyphenolic flavonoids with backbone of 3-hydroxy-2-phenylchromen-4-one (Figure 13). Their skeletons have 3-hydroxyflavone. They have double bond between position C2 and C3 and a ketone group at position C4. Unlike flavones, flavonols have hydroxyl group at C3, and sometime can link with sugar moiety, which can be glycosylated [37,38,39,40]. This sugar can be either rhamnose or glucose, but sometimes it could be galactose, xylose, arabinose and glucuronic acid. Their glycosylated form mostly found in fruits, vegetables and plant-derived foods. Mostly, flavonols are represented by glycosides of myricetin, kaempferol, quercetin and isorhamnetin [37,38,39,40]. Table 2 shows structure activity relationship of some isolated flavonols having anti-depressant action.

**Figure 14:**
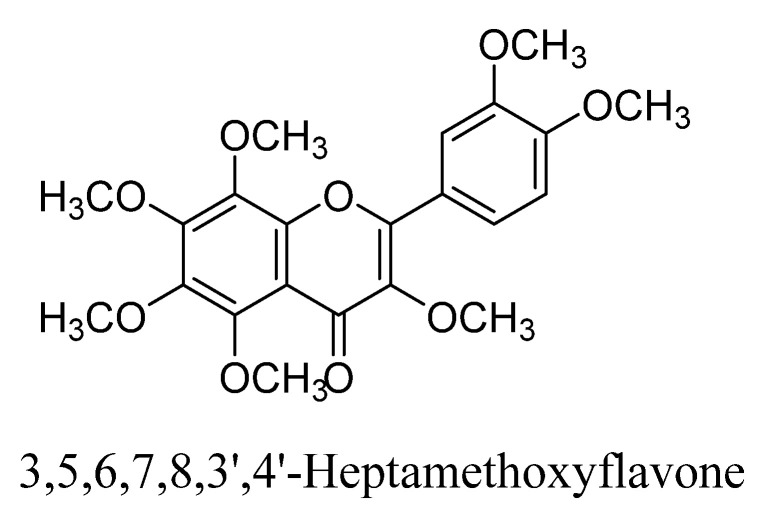
3,5,6,7,8,3′,4′-heptamethoxyflavone.

Further, 3,5,6,7,8,3′,4′-heptamethoxyflavone (Figure 14) is a citrus flavonoid derived from citrus fruits [78]. This flavonol exhibited several pharmacological activities such as neuroprotective, immune-modulatory and anti-inflammatory activities [78,79]. According to Sawamoto et al., 3,5,6,7,8,3′,4′-heptamethoxyflavone derived from orange oil has shown antidepressant action at dose of 50 mg/kg in ischemic mice using animal model of corticosterone induced depression. This flavone found to increase the regulation of extracellular signal-regulated kinase1/2 and phosphorylation of calcium calmodulin dependent protein kinase-II. Therefore, 3,5,6,7,8,3′,4′-heptamethoxyflavone ameliorated corticosterone-induced depressive behavior and reduction in weight loss, neurogenesis, hippocampal BDNF production and expression in mice [80].

**Figure 15:**
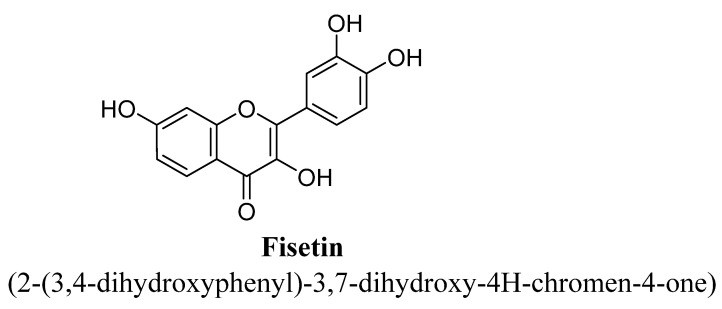
Fisetin.

Fisetin is a 7, 3′, 4′-flavon-3-ol (Figure 15) bioactive flavonoid and highly abundant in vegetables and fruits, especially in strawberries. This flavonoid has been found to exert various pharmacological activities, including anti-inflammatory, antioxidant and neuroprotective actions [81,82]. Zhen et al., have proven the antidepressant action of fisetin in rodents and suggested the involvement of noradrenergic and serotonergic systems. Even the author confirmed that the inhibitory action of fisetin on monoamine oxidase enzyme, i.e.**,** also contributed to its anti-depressant action [83]. Further, Yu et al., have also demonstrated anti-depressant potential of fisetin and found to reverse depressive behaviour in a lipopolysaccharide-(LPS-) induced acute neuro-inflammation animal model, which confirmed its potential to become a potent candidate for neurological and psychological disorder therapy [84]. In another study, the therapeutic potential of fisetin for treating depression was found to be mediated via activation of TrkB signaling pathway and leads to increase in phosphorylation of TrkB level without disturbing total TrkB [85].

**Figure 16:**
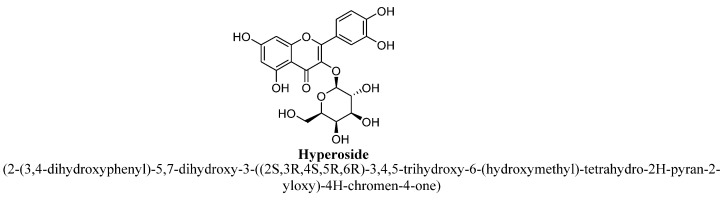
Hyperoside.

Hyperoside (tetrahydroxyflavone) is a natural flavonol and also known as 3-O-galactoside of quercetin, in which β-D-galactosyl residue attached at C3 (Figure 16) [86]. In 2012, Zheng et al., has reported that hyperoside isolated from hydroethanolic extract of *Apocynum venetum* L. leaves, exhibited antidepressant effects and has shown cytoprotective action via upregulation of CREB (cAMP response elements binding protein) and BDNF expression through regulating AC-cAMP-CREB signalling pathway [87]. In 2011, Haas et al., have also proved the anti-depressant potential of hyperoside isolated from methanolic extract of aerial part of *Hypericum caprifoliatum* L., which was claimed to be mediated via activation of D2-DA receptors of dopaminergic system [88]. Another study has also shown its antidepressant potential mediating via modulation of HPA axis by reducing plasma corticosterone and ACTH levels [89].

**Figure 17:**
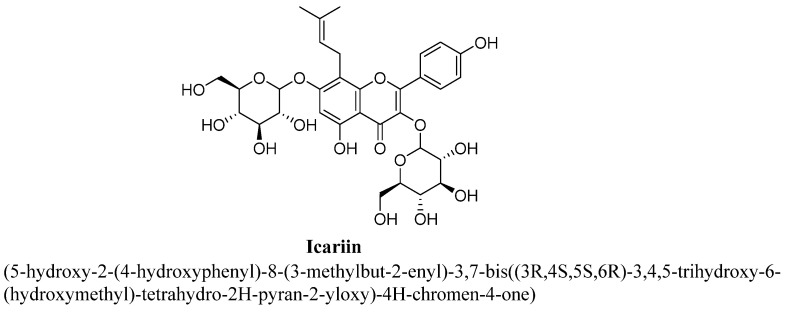
Icariin.

Icariin is an 8-prenyl derivative of kaempferol 3, 7-O-diglucoside and also known as prenylated flavonol glycoside (Figure 17) [90]. It is highly found in *Herba epimedii*, which is a traditional Chinese herb and used for treating from centuries. Icariin was found to possess significant neuroprotective and anti-depressant actions evaluated using chronic stress induced animal models [91]. Wu et al., has studied that icariin helps in partly restoring of social defeat induced impairment of HPA axis hyperactivity and glucocorticoid sensitivity. This result in normalization of glucocorticoid receptors function and also increase in hippocampal BDNF level and expression [92]. Further, Liu et al., and Wei et al., have also confirmed anti-depressant effect of icariin using unpredictable chronic stress-induced depression model of rodents and found to decrease hippocampal neuroinflammation, inhibited inducible nitric oxide synthase enzyme activity via acting on different targets in prefrontal cortex and hippocampus [91]. According to Wei et al., icariin has found to decrease in levels of SGK1 (serum and glucocorticoid-regulated kinase 1) and FKBP5 (FK506 binding protein 5) expression and restore negative feedback regulation of HPA axis via normalizing gluco-corticoids receptor [93]. Gong MJ et al., revealed anti-depressant potential of icariin using corticosterone- induced depression model and concluded that this effect was mediated via increase in BDNF level and regulation of metabolic dysfunction and pathways [94].

**Figure 18:**
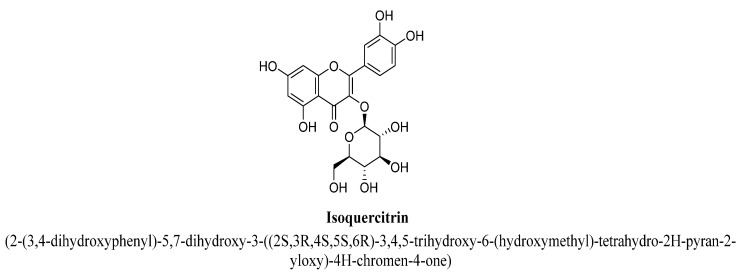
Isoquercitrin.

**Figure 19:**
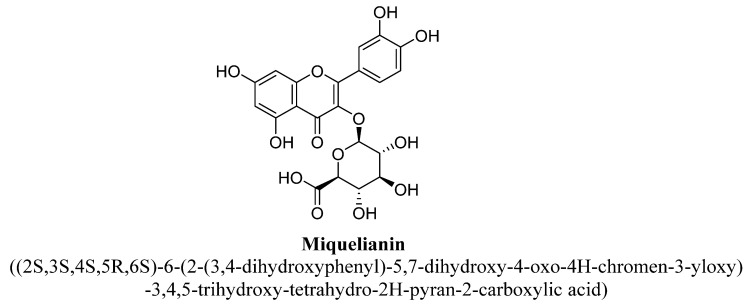
Miquelianin.

Isoquercitrin is a 3-O-glucoside of quercetin (Figure 18) and known as isoquercetin and isotrifoliin. In a study, isoquercitrin, miquelianin and hyperoside isolated from methanolic extract of aerial parts of St. John’s wort found to exhibited anti-depressant action in rats acting via modulating functions of HPA axis (hypothalamic–pituitary–adrenal axis) by preventing hyper secretion of cortisol and adrenocorticotropic hormone [89]. Scheggi S et al. has demonstrated the anti-depressant potential of isoquercitrin, quercetin and rutin isolated from methanolic extract of aerial parts of *Hypericum connatum* L. in rats, which may be due to their antioxidant potential [95]. Miquelianin is a quercetin 3-O-glucuronide (Figure 19) and also found in green beans, wine or *Nelumbo nucifera*. It is found to be presented in plasma or urine as metabolite of quercetin, tea and cocoa, and exert strong antioxidant action in the body [96].

**Figure 20:**
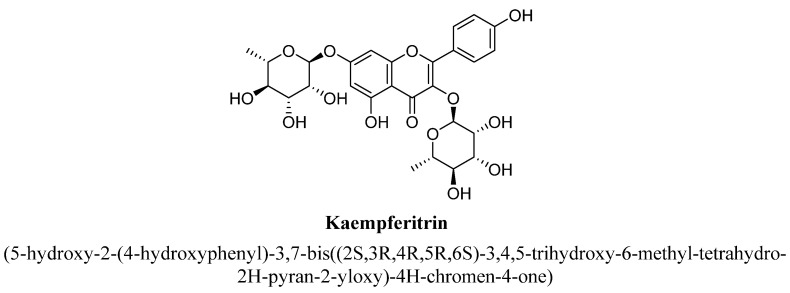
Kaempferitrin.

Kaempferitrin is a 3, 7-dirhamnoside of kaempferol (Figure 20) and extracted from various plants [97]. Cassani et al., has reported that kaempferitrin isolated from hydroethanolic extract of aerial parts of *Justicia spicigera* Schltdl plant exhibited anti-depressant action in animal behavioral models [98]. In addition, this plant has been reported to possess various pharmacological properties, i.e., ant-inflammatory, antidiabetic, anti-seizure and analgesic actions [98]. The antidepressant potential of kaempferitrin was suggested to be mediated through serotonergic system (mainly, presynaptic 5-HT1A receptors) and also via regulating the HPA axis [98].

**Figure 21:**
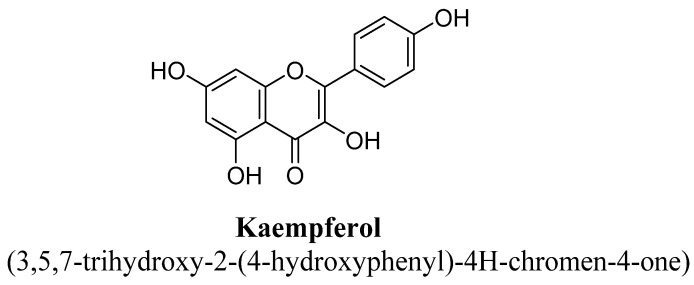
Kaempferol.

**Figure 22:**
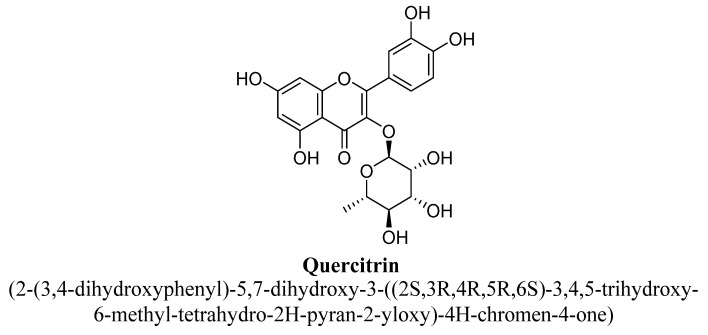
Quercitrin.

**Figure 23:**
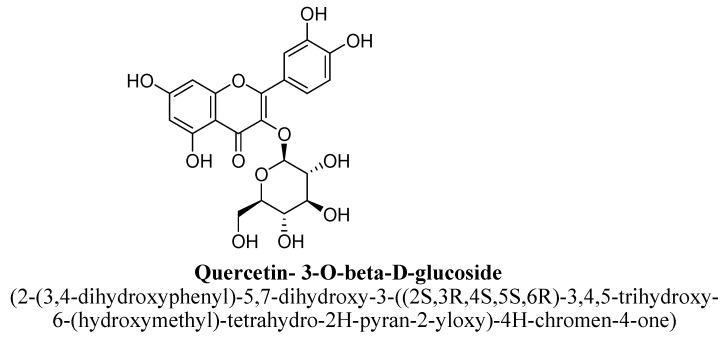
Quercetin-3-O-beta-D-glucoside.

**Figure 24:**
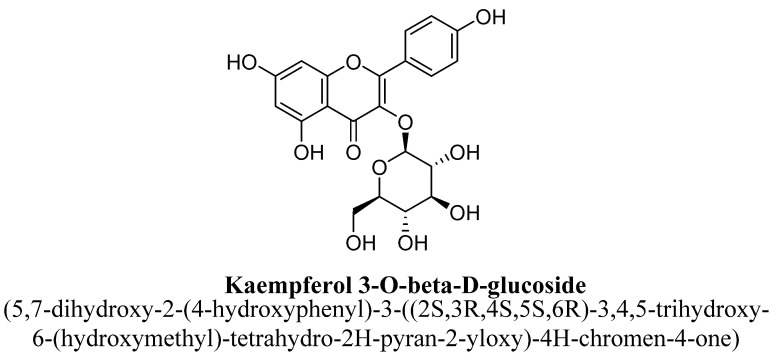
Kaempferol-3-O-beta-D-glucoside.

Kaempferol is a 3, 4′,5,7-tetrahydroxyflavone consisting of the hydroxyl group at positions C3, C5, C7 and C4′ (Figure 21). It has been found in varieties of plants, fruits and vegetables such as tea, broccoli, tomatoes, *Ginkgo biloba* L. and grapes, etc. [99]. In a pre-clinical study, kaempferol and quercetrin isolated from hydroethanolic extract of aerial parts of *Opuntia ficus-indica* indica var. saboten were found to exhibit anti-depressant action in tail suspension and forced swim test and the underlying mechanism was found to be mediated via increasing level of plasma β-endorphin or POMC mRNA in mice [100]. Quercetrin (tetrahydroxyflavone) is a quercetin O-glycoside, in which quercetin is substituted with α-L-rhamnosyl moiety at position C3 via glycosidic linkage (Figure 22) [100]. In another study, kaempferol, kaempferol-3-O-β-D-glucoside, quercetin, and quercetin3-O-β-D-glucoside (Figure 23) isolated from hydroethanolic extract of *Apocynum venetum* L. leaves, were also found to exert significant anti-depressant actions in mice [101]. The underlying mechanism behind this effect was suggested to be increase in dopamine, serotonin and nor-epinephrine level with reduction in serotonin metabolism [101]. Kaempferol-3-O-β-D-glucoside is a trihydroxyflavone, which is also known as kaempferol O-glucoside and having glucosyl residue at position C3 of kaempferol via a β-glycosidic linkage (Figure 24). It is mainly present in red wine and having strong antioxidant activity. Quercetin3-O-β-D-glucoside is a quercetin O-glucoside, in which quercetin have β-D-glucosyl residue attached at position C3, derived from β-D-glucose [101].

**Figure 25:**
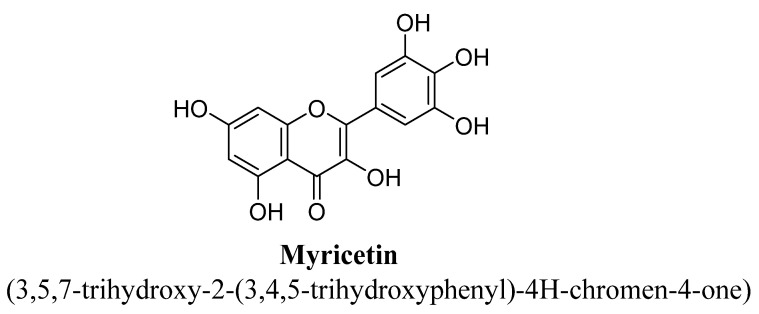
Myricetin.

Myricetin is a hexahydroxyflavone and substituted with hydroxyl group at C3, C3′, C4′, C5, C5′ and C7 (Figure 25). It is highly abundant in fruits, vegetables, nuts, red wine, berries, and tea. It has been documented for anti-inflammatory, antioxidant, neuroprotective and anti-apoptotic properties [102,103]. In a study, myricetin has found to decreased depressive behavior in mice when exposed to stress, which was predicting in forced swim and tail suspension test. This flavonol has found to decreased plasma level of corticosterone, improved activity of glutathione peroxidase enzyme in hippocampus and also increased BDNF level, all these results contributed to anti-depressant potential of myricetin [104].

**Figure 26:**
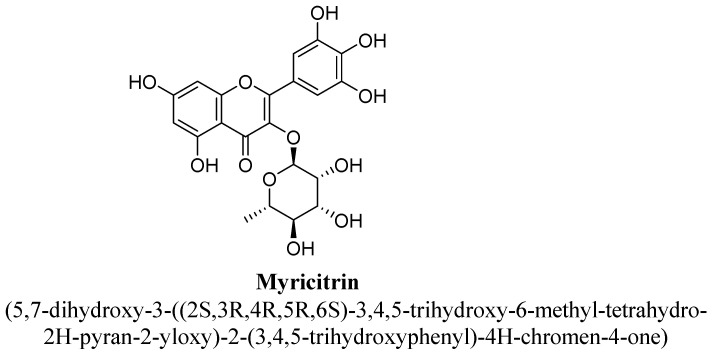
Myricitrin.

Myricitrin (glycosyloxyflavone or pentahydroxyflavone) is a 3-O-α-L-rhamnopyranoside of myricetin in which myricetin is attached to α-L-rhamnopyranosyl residue at position C3 via glycosidic linkage (Figure 26) [105]. In literature, it has been found to possess antioxidant, anti-fibrotic and anti-inflammatory activities [105]. Pre-clinically, it has shown potent anti-depressant potential in tail suspension test using mice. This effect was proposed to be mediated through inhibition of nitric oxide and hippocampal neurogenesis [106]. In addition, myricetin increases neuronal proliferation, growth and their survival, especially in the subventricular zone and the subgranular zone [106].

**Figure 27:**
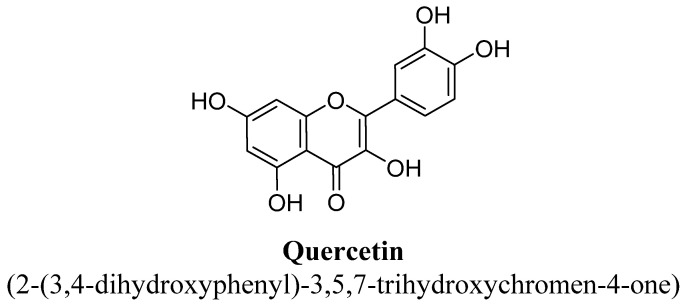
Quercetin.

Quercetin is a pentahydroxyflavone (Figure 27) and highly abundant in apple, onion, broccoli, wine and plants such as green tea and *Ginkgo biloba* [107]. This flavonol has been documented to have significant free radical scavenging property, which helps in amelioration of various diseases and disorders [107]. In addition, anti-depressant potential has also been proven in various animal models and found to increase synaptic cleft serotonin and nor-epinephrine availability via inhibition of MAO enzyme [50,101]. Demir et al., has demonstrated antidepressant potential of quercetin in diabetic rodents and has concluded that it could be considered as a potent supplement for treating depression in diabetic condition [108]. In addition, Rinwa and Kumar have reported that it produced antidepressant effect in olfactory bulbectomized rats and also suppresses micro-glial neuro-inflammation in rat brain [109].

**Figure 28:**
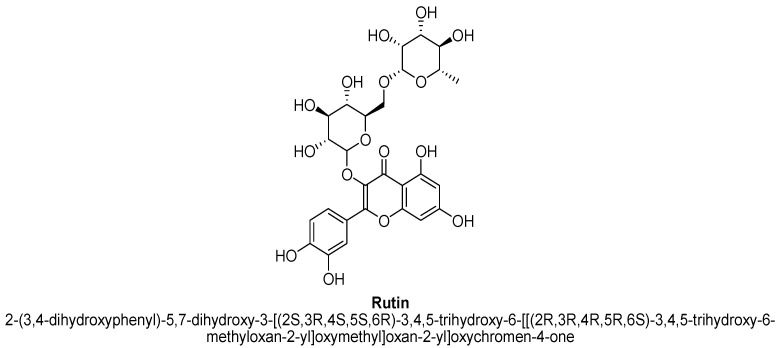
Rutin.

Rutin (tetrahydroxyflavone) is a citrus bioflavonoid (Figure 28), which is also known as quercetin-3-O-rutinoside, sophorin and rutoside [110,111,112]. It is the glycoside combination of flavonol quercetin and disaccharide rutinose, having quercetin with -OH at position C3 substituted with rhamnose and glucose sugar groups. It has been found in citrus fruits, vegetables and plants, such as black tea, green tea, figs and buckwheat, etc. [110,111]. It helps in producing collagen and usage of vitamin C in body. It has been documented to possess several pharmacological actions, i.e., it is neuroprotective, antioxidant, anti-inflammatory and anti-tumor, etc. [110,111,112]. According to Machado DG et al., report, rutin isolated from ethanolic extract from aerial parts of *Schinus molle* L. has shown antidepressant action in forced swim and tail suspension test. Increases in the level of synaptic nor-adrenaline and serotonin justified the anti-depressant action of rutin [8].

#### SAR of Flavonols

**Figure 29:**
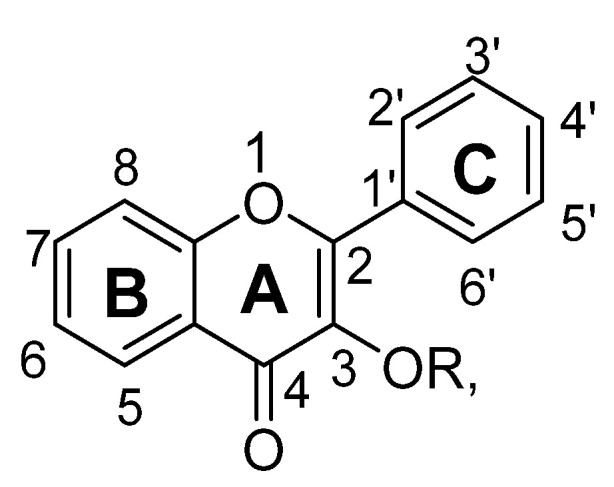
SAR of Flavonols.

Based on the information available on the activities of various flavonols, the following SAR appears to be applicable (Figure 29).

1. In flavonols, attachment at C3 with –OR, does not much affect the antidepressant potential.
2. Substitution at C3 with hydroxyl group resulting in increase of brain levels of neurotransmitter, i.e., serotonin, dopamine, nor-epinephrine.
3. The presence of ketonic group at C4 and ring B, C may be necessary for anti-depressant potential of flavonols.
4. Substitution at –OR at C3 with long chain molecules (C6H11O5 or C6H11O6) resulting in decrease activation of hypothalamic-pituitary-adrenal axis, thereby decrease the release of ACTH.
5. Substitution of –OR at C3 with long chain molecule may be responsible for decrease in BDNF activity of flavonols.
6. Substitution of –OR with –OH group decreases the MAO inhibitory potential.
7. Hydroxyl group at C3′ and C4′ is necessary for increment of antioxidant potential and radical scavenging property.
8. Acetate and methyl group at C7 and C8 decreased antioxidant potential of flavones.
9. Glycoside-O linkage at C7 abolished or reduces the MAO inhibitory potential.

### 2.3. Flavanones

**Figure 30:**
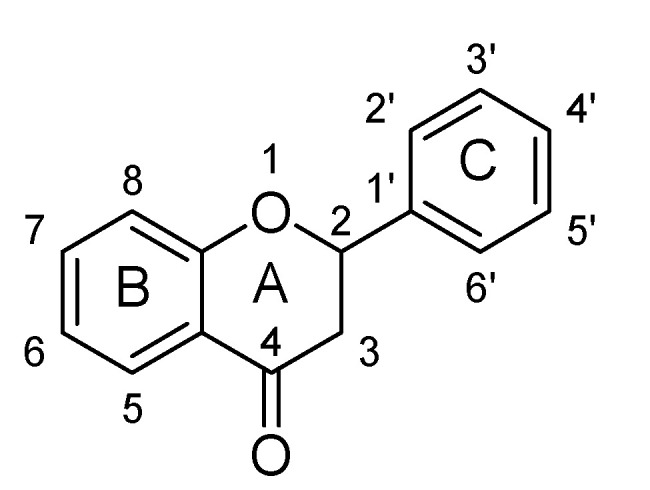
Skeleton of Flavanones.

Flavanones are derived from hybrid of flavanes and also known as 2-phenyl-2, 3-dihydrochromen-4-one (Figure 30). Structurally, flavanones consist of flavan having oxo substituent at position C4. They are highly abundant in citrus vegetables and fruits such as grapes, tomato and cherries etc. Unlike flavones, flavanones do not have double bond between position C2 and C3 [37,38,39,40]. Flavanones possess very strong antioxidant activity, which helps in curing various oxidative-stress related maladies such as cardiovascular disease, central nervous system disorders, cancer and atherosclerosis [38,39,40]. Their pharmacological properties include anti-inflammatory, antioxidant, antimicrobial and antiviral activities [37,38,39,40]. In recent years, several flavanones have been isolated and found to reduce depressive-like behavior when evaluated for anti-depressant action in rodents (Table 3).

**Figure 31:**
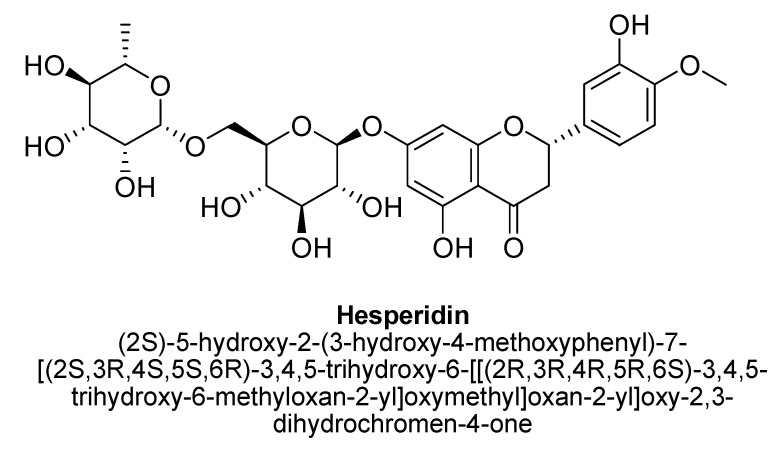
Hesperidin.

Hesperidin (3′-hydroxyflavanones) is a natural bioflavonoid predominant in citrus vegetables and fruits. Structurally, it composed of hesperetin substituted with a 6-O-(alpha-L-rhamnopyranosyl)-beta-D-glucopyranosyl moiety at position C7 via glycosidic linkage (Figure 31). It has been reported to possess multiple therapeutic properties, i.e.**,** antioxidant, antidiabetic, antineoplastic, neuroprotective and anticancer properties, which have been evaluated in vivo and in vitro [113,114,115,116]. El-Marasy et al. studies the antidepressant potential of hesperidin using streptozotocin-induced diabetic rat model. The authors suggested multiple underlying mechanisms responsible for anti-depressant property via modulating hyperglycemia also, i.e.**,** increases in monoamine level in brain, induction of hippocampal BDNF level and expression, anti-inflammatory and antioxidant activities [117]. In another study, hesperidine at doses 0.1, 0.3, and 1 mg/kg (i.p.) has shown antidepressant potential in Swiss albino mice as it reduced the immobility time in mice model [118]. Increase in activation of serotonergic, noradrenergic, and dopaminergic systems, action at receptor 5-HT1A and increase in hippocampal BDNF concentrations were found to be responsible for antidepressant-like effect of these flavanones [118,119]. Moreover, hesperidin has been found to decrease reactive oxidative species (ROS) generation, malondialdehyde (MDA) formation, enhances glutathione levels and superoxide dismutase in human cell lines, i.e., ARPE-19 and HaCaT cells [118,119,120]. In addition, in-vitro and animal models, quercetin, naringenin, astilbin and hesperidin were found to prevent depressive symptoms because of their antioxidant potential, defence response against inflammatory cascades and monoamine oxidases inhibitory action [30]. Furthermore, Li et al., evaluated the antidepressant potential of hesperidin in mice using chronic mild stress rodent model that results in amelioration of the reduction in sucrose preference and also, revert immobility time of mice induced by chronic mild stress. The outcome of this study claimed the involvement of extracellular signal regulated kinase-(ERK-) BDNF signaling pathway in the antidepressant action of hesperidine [121]. Donato et al., also observed that administration of hesperidin combats the depressive-like behaviors in mice via inhibition of L-arginine-NO-cGMP pathway, and increasing in hippocampal BDNF expression and its level [122].

**Figure 32:**
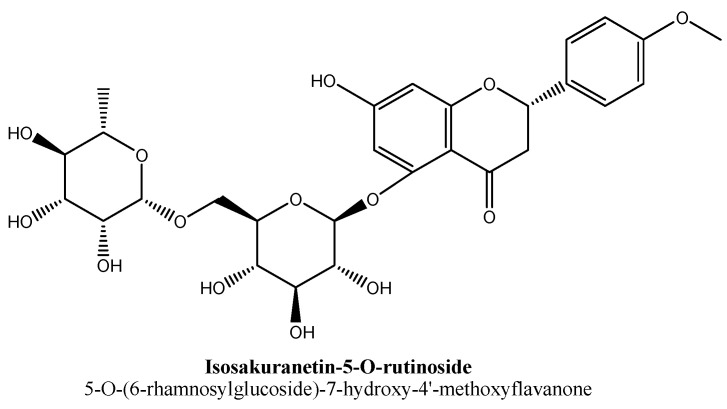
Isosakuranetin-5-O-rutinoside.

Isosakuranetin-5-O-rutinoside is a new flavonoid and also known as 5-O-(6-rhamnosylglucoside)-7-hydroxy-4′-methoxyflavanone (Figure 32). This flavanone was isolated from hydroethanolic extract of leaves of plant *Salvia elegans* Vahl and found to possess antidepressant activity in animal model, i.e.**,** forced swimming test [123]. Further, reports on underlying anti-depressant mechanism of this new flavanone are not available.

**Figure 33:**
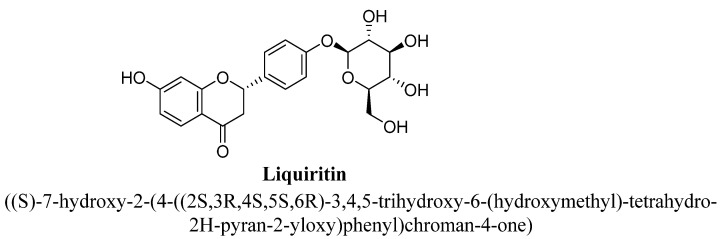
Liquiritin.

**Figure 34:**
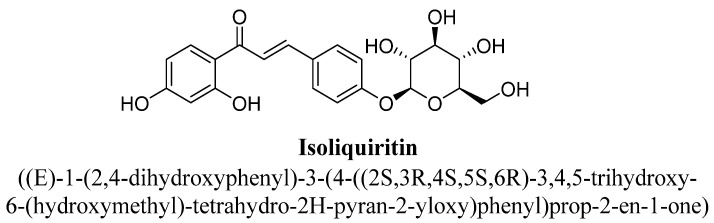
Isoliquiritin.

Liquiritin is a 4′-O-glucoside of the flavanone liquiritigenin, in which a β-D-glucopyranosyl residue is attached to liquiritigenin at position C4′ by a glycosidic linkage (Figure 33) and found in spices and herbs. Whereas isoliquiritin is a trans-chalcone derivative, C2′ and C4′ positions substituted with hydroxyl group and a β-D-glucopyranosyloxy moiety attached at position C4 (Figure 34). liquiritin and isoliquiritin isolated from aqueous extract of roots of *Glycyrrhiza uralensis* Fisch. were evaluated for their anti-depressant potential in mice at doses 10, 20 and 40 mg/kg using forced swim and tail suspension test [124]. Further, these flavonoids found to produce significant antidepressant effect and their proposed mechanism includes slowing down of 5-HT metabolism, significant reduction in 5-HIAA/5-HT ratios and increased concentration of monoamine neurotransmitters (especially, serotonin and nor-epinephrine) in the cortex, hypothalamus and hippocampus of mice [124].

**Figure 35:**
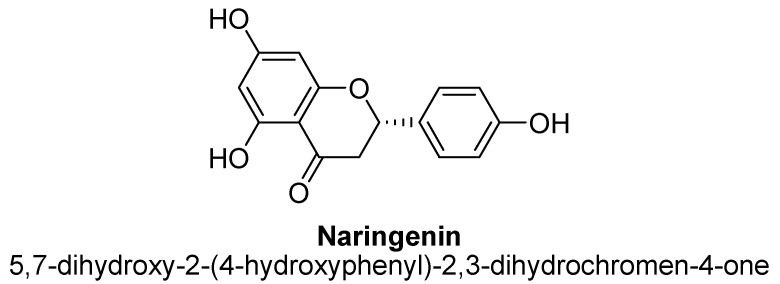
Naringenin.

Naringenin is a trihydroxyflavanone and highly prevalent in citrus fruits peel. Structurally, it is substituted with three hydroxy groups at positions C5, C6 and C4′ (Figure 35). It has been documented to possess multiple therapeutic properties such as anti-inflammatory, antioxidant, neuroprotective, cognition enhancing, MAO inhibitor, anti-bacterial and wound-healing [125,126,127]. Likewise, it was found to possess antidepressant effects also. In addition, it has been found to stimulate monoamines and suppress neuro-endocrine signaling and, which leads to up-regulation of hippocampal BDNF in rodents [128,129]. In another study, naringenin isolated from methanolic extract of citrus peel, found to reduce immobility duration in tail suspension animal model of mice at dose of 5, 10 and 20 mg/kg, which interpreted it as potent antidepressant action [130]. This effect was suggested to be involved the activation of noradrenergic and serotonergic monoamine systems in mice brain [130]. In addition, naringenin at doses of 10 and 20 mg/kg found to increase BDNF expression in the hippocampus of mice [128].

**Figure 36:**
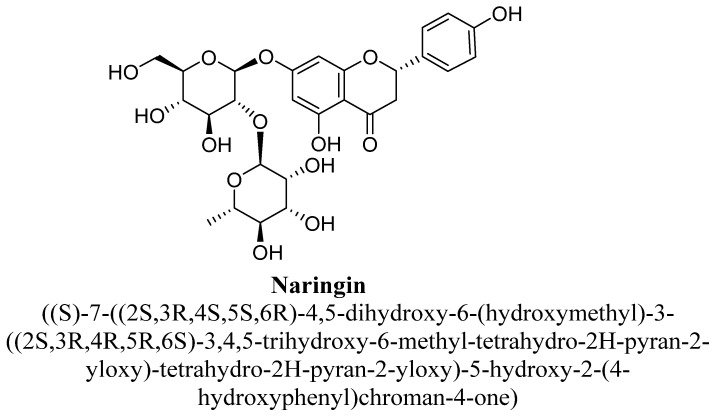
Naringin.

Naringin (4′-hydroxyflavanones) is a flavanone-7-O-glycoside (Figure 36) and occur naturally in citrus fruits especially in grapes. It is responsible for bitter taste of fruits. Structurally, it is (S)-naringenin substituted with a 2-O-(α-L-rhamnopyranosyl)-β-D-glucopyranosyl moiety at position C7 by a glycosidic linkage. It exerts various pharmacological actions such as anticarcinogenic, blood lipid-lowering, antioxidant activity and inhibition of cytochrome P450 enzymes (CYP1A2 and CYP3A4) [131,132]. Kwatra M et al., studies that naringin and sertraline significantly prevent doxycycline-induced depression and anxiety in mice evidenced from forced swimming test and elevated plus maze. Decrease in hippocampal tumor necrosis factor-α, interleukin-1 β and plasma corticosterone levels were observed in naringin, and sertraline treated mice. Furthermore, their combination found to diminished hippocampal oxidative stress, modulated mitochondrial-complexes protection pathway and 5-HT levels [133].

#### SAR of Flavanones

**Figure 37:**
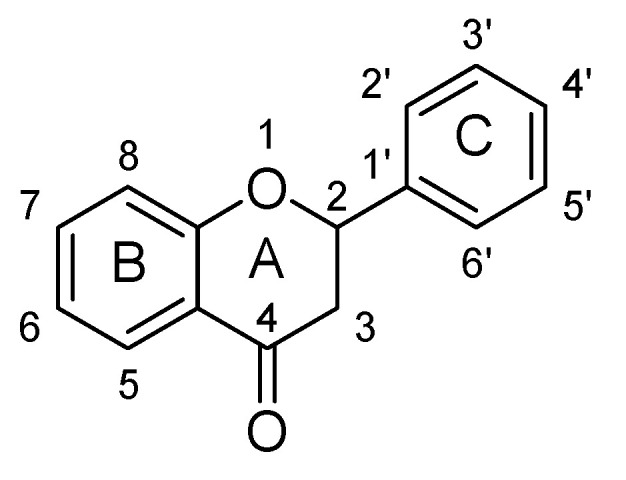
SAR of Flavanones.

Based on the information available on the activities of various flavanones, following SAR appears to be applicable (Figure 37).

1. In flavanones, there is absence of double bond at C2 and C3 position; it means this bond is not necessary for antidepressant potential.
2. Saturation of double bond at C2 and C3 position does not much affect the BDNF activity of flavanones.
3. Saturation of double bond at C2 and C3 position reduces the MAO inhibitory potential.
4. Substitution at C7 with O- (C12H21O9) may be responsible for selective interaction with kappa-opioid receptors.
5. Glycoside-O linkage at C7 abolished or reduces the MAO inhibitory potential.
6. Acetate and methyl group at C7 and C8 decreased antioxidant potential of flavonols.
7. Hydroxyl group at C3′ and C4′ is necessary for an increment of antioxidant potential and radical scavenging property.
8. Hydroxyl group at C3′ may be responsible for reduction in acetyl-cholinesterase activity.

### 2.4. Flavanonols

**Figure 38:**
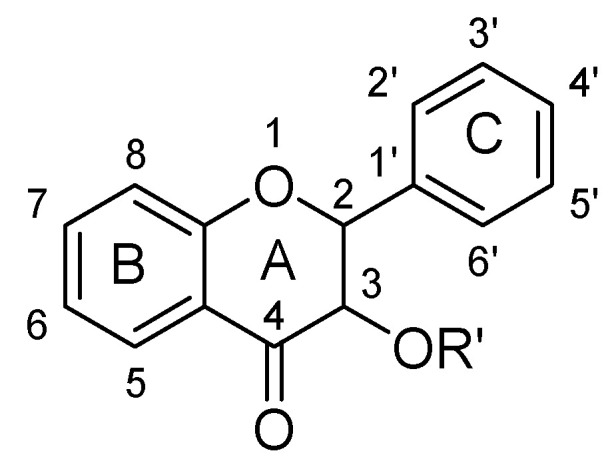
Skeleton of Flavanonols.

Flavanonols have backbone of 3-hydroxy-2, 3-dihydro-2-phenylchromen-4-one. Basically, they are 2-phenyl-3, 4-dihydro-2H-1-benzopyran having a ketone and a hydroxyl group at C4 and C3 as shown below (Figure 38) [37,38,39,40]. Astilbin and dihydromyricetn were found to possess anti-depressant action, which are analogues of flavanonols as shown in (Table 4).

**Figure 39:**
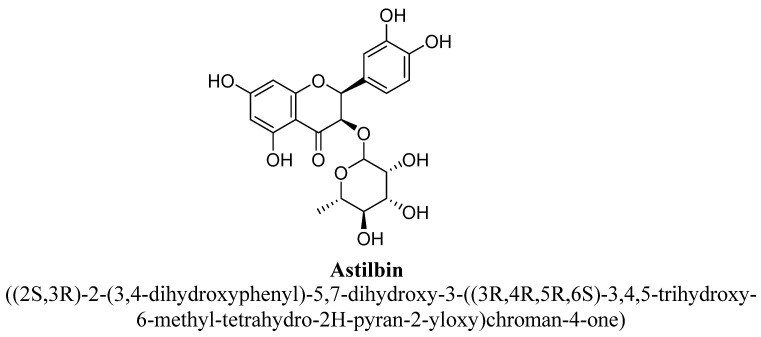
Astilbin.

Astilbin is a natural flavonoid, structurally composed of (+)-taxifolin substituted by a α-L-rhamnosyl moiety at position C3 by a glycosidic linkage (Figure 39). It is highly abundant in plants of *Hypericum perforatum* and has various pharmacological actions such as anti-inflammatory function, free radical scavenging and antioxidant properties [134,135]. Lv et al., studies the antidepressant potential of astilbin and suggested that this effect was found to mediate via up regulation of BDNF signaling pathway and neurotransmitters discharge in mice cortex by inhibiting MAO enzyme [136].

**Figure 40:**
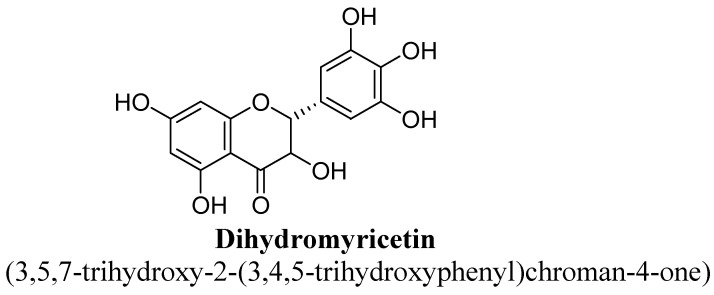
Dihydromyricetin.

Dihydromyricetin is a flavanonols, structurally hydroxyl group present at C3, C5, C6, C3′, C4′ and C5′ (Figure 40). It also known as Ampelopsin and widely found in the *Ampelopsis* species *grossedentata*, *megalophylla*, *japonica*; *Hovenia dulcis*; *Cercidiphyllum japonicum*; *Rhododendron cinnabarinum*; *Pinus species*; *Salix sachalinensis*; and *Cedrus* species. Ren Z et al., observed that dihydromyricetin has significant anti-depressant action which was investigated using experimental animal models and found to be associated with inhibition of neuro-inflammation and increase in BDNF expression [137].

### 2.5. Flavanols

**Figure 41:**
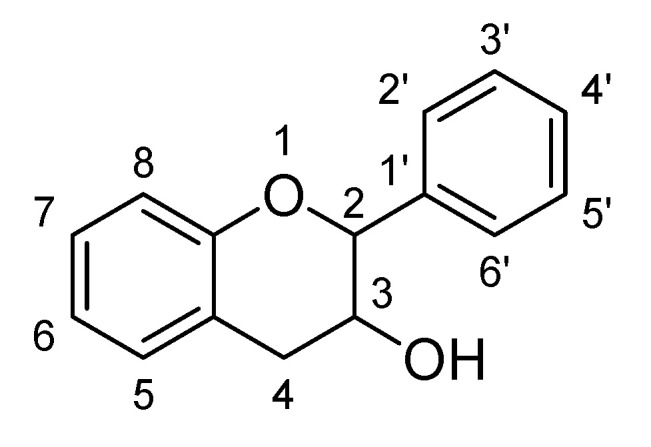
Skeleton of Flavanols.

Flavanols, chemically defined as flavan-3-ols, are one of the most important subclasses of flavonoids (Figure 41). They are also commonly known as catechins. Structurally, there is absence of a ketone group at C4 and a double bond between C2 and C3, results in presence of two chiral carbons in flavonols. Hence, there are four possible diastereomers present i.e., (−)-catechin (2S,3R), (+)-catechin (2R,3S), (−)-epicatechin (2S,3S) and (+)-epicatechin (2R,3R). Flavanols are commonly found in berries and grapes in the form of (−) –epicatechin and (+) –catechin, whereas epigallocatechin and epicatechingallate and have been found to be present in tea [138].

**Figure 42:**
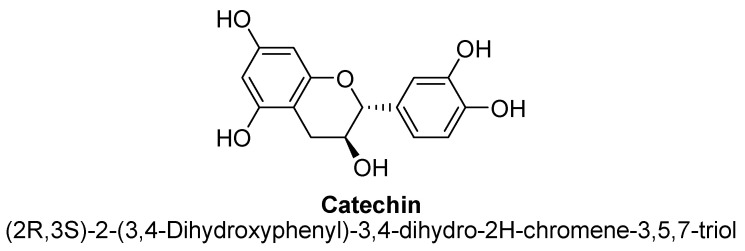
Catechin.

**Figure 43:**
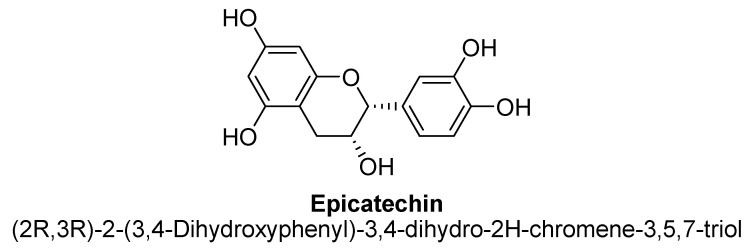
Epicatechin.

The flavan-3-ols (−)-epicatechin and (+)-catechin isolated from methanolic extract of dried hooks of *Uncaria rhynchophylla* (Miq.). Jacks have shown significant protective activity against neurodegeneration via inhibiting MAO-B enzyme activity in rodent brain, thus found to treat the symptoms of anxiety and depression [139]. Cocoa and dark chocolate having a cocoa content of 70% or more are widely known for their action on symptoms related to depression. Cocoa has been found to contains (+)-catechin and (−)-epicatechin (Figure 42 and Figure 43). In a study, polyphenolic extract of cocoa, containing high levels of flavanols, has been found to exhibit antidepressant-like action in mice evaluated using forced swim test paradigm [140].

**Figure 44:**
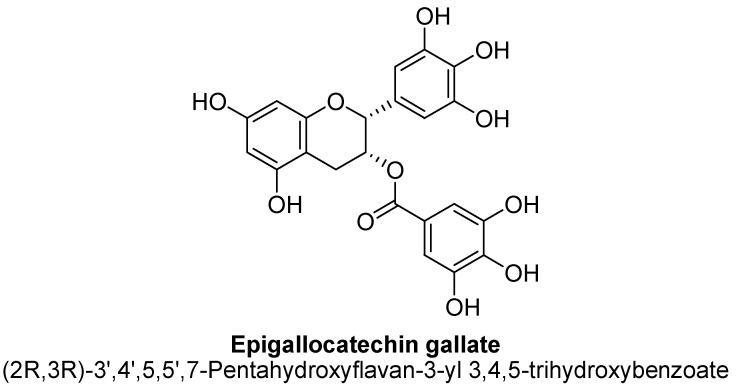
Epigallocatechin gallate.

Recently, Zafir et al., have proved that chronic administration of various antidepressants to mice has caused a decrease in the activity of antioxidant enzymes, such as catalase and superoxide dismutase and also, reduced the normalization of lipid peroxidation. In contrast, flavonols have been found to exhibit strong antioxidant activity [141]. Li et al., have proved that epigallocatechin gallate (Figure 44) and catechin reduces the astrocyte and/or microglia mediated neuro-inflammation, particularly by inhibiting the release of cytokine. All of these concomitant data have strongly suggested that flavanols, with their strong antioxidant activity, regulates oxidative stress, better than well-established synthetic antidepressant, whereas, their role in the central nervous system is complicated, specially, in the relationship with the modulation of genetic expression, mitochondrial neuro-inflammation and intracellular signaling cascades [142].

### 2.6. Others

**Figure 45:**
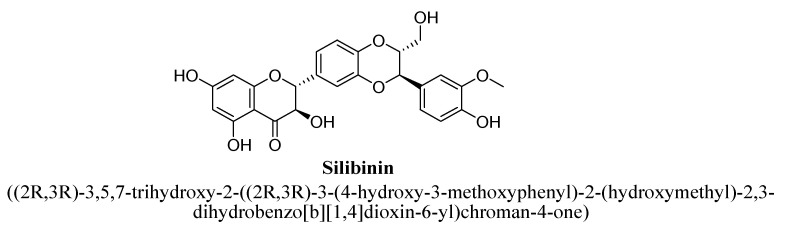
Silibinin.

Silibinin (Figure 45) is the major bioactive constituent of silymarin (containing a mixture of flavonolignans composed of isosilibinin, silibinin, silidianin, silychristin, etc.) extract prepared from *Silybum marianum* (i.e., milk thistle) seeds. It has been reported to possess pharmacological activities such as antioxidant, hepatoprotective, cardioprotective, anti-inflammatory and anticancer activities [133]. It has been found to exert anti-depressant action in rats via altering immunological, endocrine and monoamines systems such as 5-HT, DA, NE, MDA formation, TNF-α, IL-6 and BDNF levels in hippocampus and cerebral cortex [143,144].

## 3. Possible Cellular and Molecular Mechanism of Anti-Depressant Action of Flavonoids

**Figure 46:**
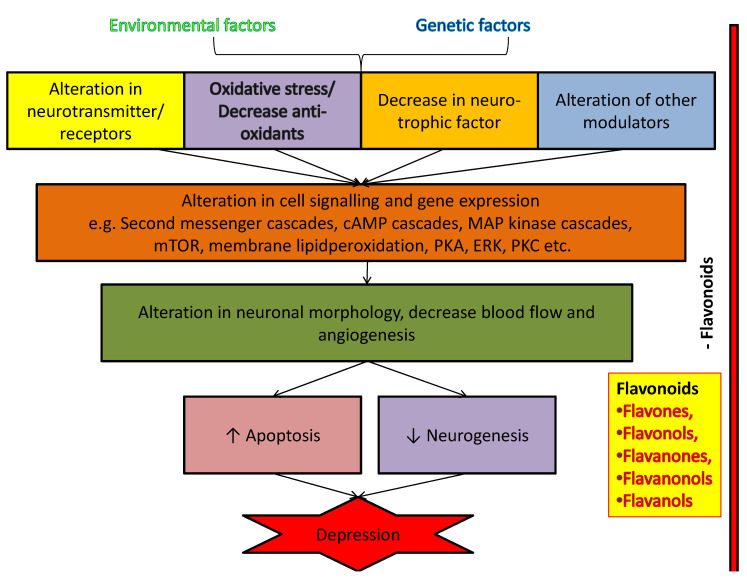
Possible anti-depressant mechanisms of flavonoids. cAMP, cyclic adenosine monophosphate; MAP, mitogen-activated protein; mTOR, mammalian target of rapamycin; PKA, protein kinase A; ERK, extracellular regulated kinase; PKC, protein kinase C.

Naturally occurring flavonoids were found to exhibit positive effects in depression with poorly understood mechanisms. Although flavonoids are generally proposed to act as antidepressant analogues via inhibiting overacted apoptosis (by modulating caspases 3 and 9, Bax and Bak proteins expression), changing behavior, levels of cytokine, inhibiting oxidative stress, and also by altering energy metabolic parameters [145]. This ability of flavonoids to influence depression is partly due to their capability to interact with molecular and as well physiological mechanism [145,146]. It is believed that the concentrations of flavonoids and their reactive metabolites to the brain are sufficient to stimulate receptors, transcription factors and kinases [147]. The evidence suggests that these flavonoids have the potential to act in many ways as the exact site of their interaction is still remains unresolved with the intracellular signaling pathway for certain phytoconstituents. Mainly, antioxidant action of flavonoids contributed to its antidepressant and neuroprotective effects [145,146,147]. Beside antioxidant action, each flavonoid found to follow one or more distinct pathways against the progression and advancement of depression through affecting neurotransmission receptors, BDNF levels, increasing neuronal growth, inhibiting certain enzymes activity, i.e., MAO and acetylcholinesterase, modulation of caspases 3 and 9, Bax, Bak and cytochrome C protein expression, modulation of calcium and potassium ions channels, maintaining brain plasticity and preventing potential dissipation of mitochondrial membrane [1,29,145]. Overall, flavonoid- induced activation of neuronal signaling and gene expression in the brain, lead to alterations of brain neurogenesis and synaptic plasticity, which ultimately affect depression as shown in Figure 46.

### 3.1. Flavonoids and Neurotransmitters

**Figure 47:**
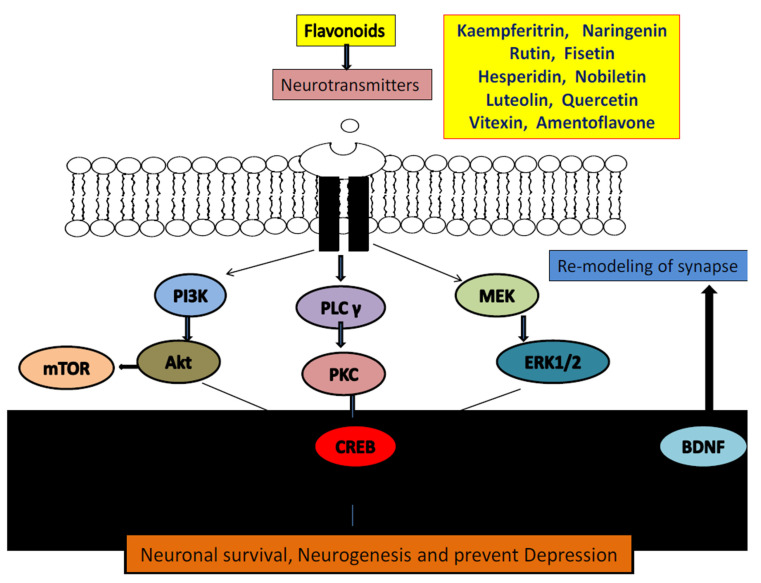
Effect of flavonoids on brain neurotransmitters**.** P13K, phosphoinositide 3-kinases; Akt (PKB), protein kinase B; mTOR, mammalian target of rapamycin; PLCγ, phospholipase C gamma; PKC, protein kinase C; MEK, mitogen-activated protein kinase; ERK1/2, extracellular regulated kinase 1 and 2; CREB, cAMP-response element binding protein; BDNF, brain-derived neurotrophic factor.

According to different monoamines hypothesis of depression, deficiencies or imbalance of monoamine neurotransmitters, i.e., serotonin, dopamine, nor-epinephrine induces the development of depressive-like symptoms [148,149,150]. The causal link between neurotransmitter and depression is the disturbances of monoamine metabolism and their receptor abnormalities. These neurotransmitters are prone to metabolism by different enzymes at each step from synthesis to binding with respective receptors. The degrading enzymes such as monoamine oxidase does metabolism of neurotransmitter after their release from vesicles [149,150,151]. It has been reported that many flavonoids possess anti-inflammatory, antidepressant and antioxidant activities in animal studies via balancing the neurotransmitters level in brain by acting at transcription factors, enzymes and kinases (Figure 47) [145,146,147,148,149,150,151,152]. Flavonoids such as rutin, amentoflavone, luteolin, nobiletin, vitexin, fisetin, kaempferitrin, quercetin, hesperidin and naringenin were found to possess anti-depressant action via modification of neurotransmitters or via acting on their pre- or post- receptors [8,45,64,66,77,83,98,101,117,120]. Whereas flavonoids such as apigenin, luteolin, oreintin, hesperidin, quercetin, fisetin and astiblin were found to inhibit MAO enzyme and increases brain neurotransmitters levels.

### 3.2. Flavonoids and Neurodegeneration

**Figure 48:**
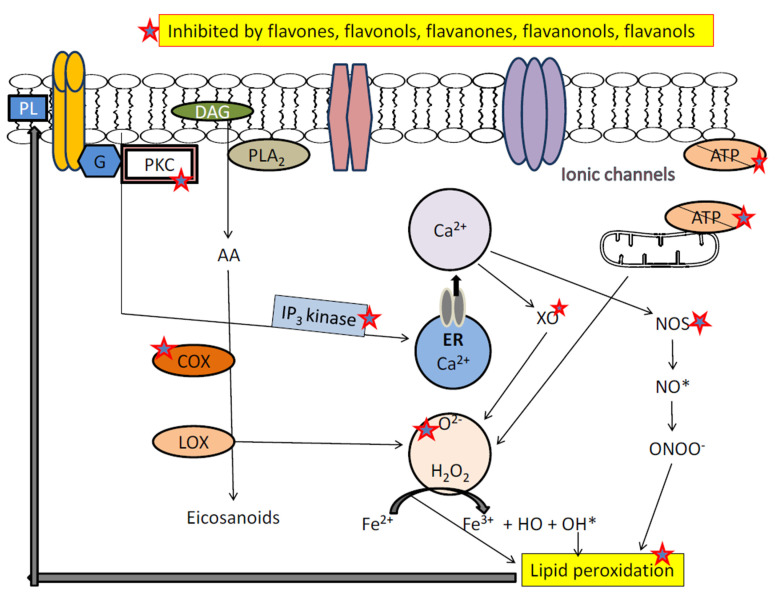
Inhibitory effect of flavonoids on lipid-peroxidation. PL, phospholipids; PKC, protein kinase C; DAG, diacylglycerol; PLA2, phospholipases A2; AA, arachidonic acid; COX, cyclooxygenase; LOX, lipoxygenases; IP3, inositol trisphosphate; ER, endoplasmic reticulum; ATP, adenosine triphosphate; NOS, *nitric oxide synthase*.

Neurodegeneration in depression is a complex phenomenon, including failure of various metabolic processes, oxidative stress, imbalance of calcium homeostasis and excitotoxicity [153,154]. In major depressive disorder, early mitochondrial metabolic failure (ATP) disrupts ionic pump function at membrane level which results in heavy release of neurotransmitters (glutamate) and increase intracellular concentrations of Na^+^ and Ca^2+^ [153,154,155]. Large-scale Ca^2+^ entry activate enzymes such as proteases, phospholipase, oxidase and endonucleases, which is responsible for hydrolysis of DNA molecule and damage the cytoskeleton [154,155,156]. Activated phospholipase A2 metabolize arachidonic acid via icosanoids and liposoxides thereby activating lipid peroxidation [153,154,155,156,157]. Increased intracellular Ca^2+^ can also activate protein kinase-C, which may modify the functions of multiple ion channels. These active intracellular metabolic events resulting in generation of the reactive oxygen radicals, which bypass antioxidant defense and provoke oxidative-stress [155,156,157,158,159]. In turn, oxidative stress provokes various changes in the macromolecules and lipid membranes, creating a vicious cycle of excessive oxidation and further oxidative damage [158,159,160]. Therefore, activation of enzymes such as xanthine oxidase (XO), cyclooxygenase (COX), nitric oxide synthase (NOS), lipoxygenase (LOX) and production of reactive oxygen and nitrogen species leads to mitochondrial DNA damage and lipid peroxidation [161,162]. In literature, several flavonoids were discovered to act in different ways in this process i.e., binds to ATP sites of receptors and enzymes, affects the function of phosphatise, modulate the activity of different kinases directly, maintain the homeostasis of calcium ion, activate transcription factor, inhibit lipid peroxidation, XO, IP3 kinase, scavenging free radicals, LOX and PKC [30,145] (Figure 48), red star shows the site of action of flavonoids).

### 3.3. Flavonoids and Oxidative Stress

**Figure 49:**
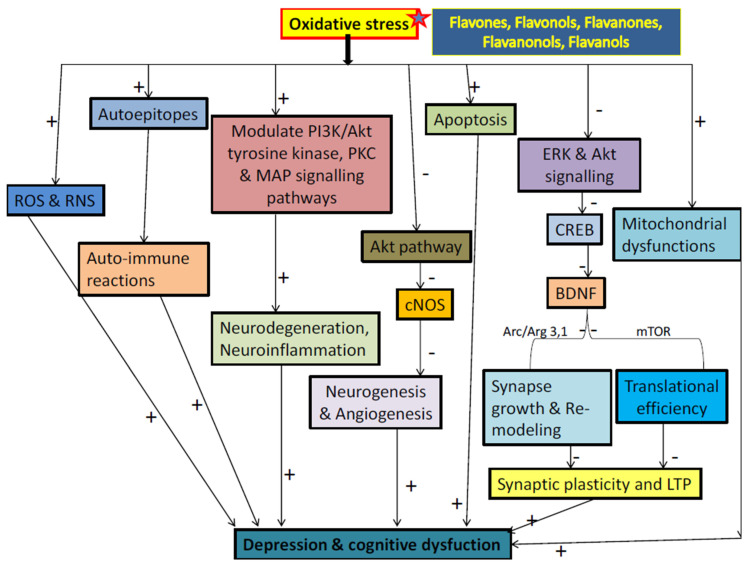
Possible antioxidant mechanism of flavonoids. ROS, reactive oxygen species; RNS, reactive nitrogen species; P13K, phosphoinositide 3-kinases; PKC, protein kinase C; MAP, mitogen-activated protein; cNOS, cyclic reactive oxygen species; ERK, extracellular regulated kinase; Akt (PKB), protein kinase B; BDNF, brain-derived neurotrophic factor; CREB, cAMP-response element binding protein; mTOR, mammalian target of rapamycin; Arc/Arg 3,1, Activity-regulated cytoskeletal-associated protein.

Oxidative stress is a biochemical imbalance between biological systems; detoxify body and reactive oxygen species, which can cause harm to organism [163]. Oxidative stress is associated with the development of much pathology, i.e., inflammatory disorders, neuronal damage, joints problem or cardio-disorder etc. [163,164]. Many compounds, including flavonoids, are classified as suppressors of oxidative stress, and therefore, are best described by their ability to be act as powerful antioxidants [165]. Catechins and flavones appear to be highly active antioxidant among all flavonoids to protect the body from reactive oxygen species [165,166]. Cells and tissues in the body are constantly damaged by reactive oxygen species and free radicals that arise during normal oxygen metabolism or are triggered by exogenous damages [163,167]. The mechanisms and sequence of events in which these free radicals interfere with function of cells are not fully understood, but lipid peroxidation is found to be most important mechanism, which result in damage to the cellular membrane. The cellular damage result in a change of overall cell charge alters osmotic pressure, causes inflammation and then cell death [163,164]. Free radicals attract several inflammatory mediators that contribute to inflammatory responses and tissues damage [168]. In order to protect them-selves from this cellular damage, organisms tend to have developed different effective mechanisms, i.e., the body’s antioxidant-defense mechanism includes enzymes such as catalase, glutathione peroxidase or superoxide dismutase, and also non-enzymatic counterparts which are tocopherol, glutathione and ascorbic acid [169,170,171]. During damage or injury, the increased level of reactive oxygen species leads in depletion and consumption of endogenous-scavenging compounds [164]. In addition, flavonoids found to have synergic and additive action with these endogenous-scavenging compounds [1,145]. Flavonoids are found to have very strong antioxidant performance, to interfere with more than three free radical producing systems at a time and eventually enhance action of endogenous antioxidants, resulting in decreases cell disruption and apoptosis [1,145] as shown in Figure 49.

In presence of iron, oxidative free radicals result in lipid peroxidation [172]. Furthermore, some flavonoids are found to chelate with iron, thereby eliminating the causative factor for free radical growth [1,145]. Quercetin is particularly known for its iron chelating and stabilizing properties [109]. Further, its other protective action is the direct protective inhibitor of lipid peroxidation [1,109].

In pathogenesis of depression, low levels of antioxidant and high level of free radicals results in lipid peroxidation, DNA strand-brakes, enzyme inactivation and neuronal damage [163,164]. Some flavonoids can remove superoxide directly, while other flavonoids refer to highly reactive oxygenated radicals known as peroxynitrite, which is a major mechanism of flavonoids [165,172,173,174,175,176,177]. The other possible underlying mechanism of flavonoids may include interaction with various enzymes functions. Therefore, flavonoids have been proven to be bioactive molecules of choice for preventing oxidative stress-induced disorder or disease such as depression [173,174,175,176]. The formation of flavonoids comprises a highly reactive hydroxyl group, which are oxidized by free radicals, resulting in a highly stable low reactive radical. These oxidative free radicals have an uneven number of electrons [173,174,175,176,177]. Therefore, flavonoids react with reactive compounds of free radical and tend to stabilize or neutralize these reactive oxidative species to prevent neuronal damage [165]. This reaction involves the following equation:

(OH) Flavonoid+ R• → (O•) flavonoid+ RH

where, R• represented as free radical, O• represented as oxidative free radical.

**Figure 50:**
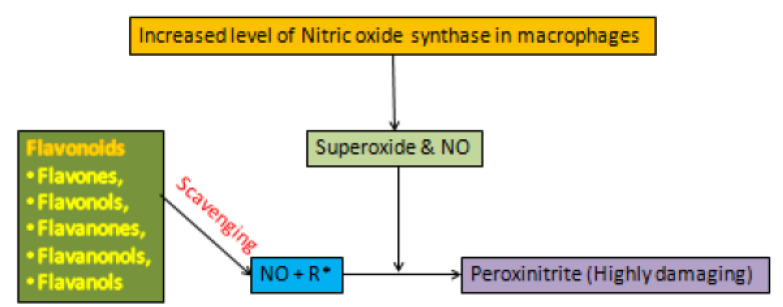
Production of nitric oxide, superoxide and peroxynitrite**.** NO, nitric oxide; R*, reactive free radicals.

Nitric oxide (NO) is produced by a variety of cells, which include macrophages and endothelial cells [178]. Increasing levels of nitric-oxide synthase in macrophages increases the production of both superoxide ion and nitric oxide. Peroxynitrite is produced when nitric oxide reacts with free radicals, which is very harmful to the cell and cause oxidative damage [178,179] as shown in Figure 50. These free radicals scavenged by antioxidant action of flavonoids and left no radical to interact with NO, resulting in minimal damage [1,145]. Even in some studies, NO itself considered as radical molecules and found to be directly scavenged by flavonoids [145].

Antioxidant enzymes such as catalase, glutathione peroxidase or superoxide dismutase (SOD) metabolizes free radicals to less toxic molecule [180]. SOD is the most important antioxidant enzyme, which catalysis the reaction of conversion of superoxide to H2O2 (comparable less toxic in nature) and also, interacted with other neuroprotective components [180,181]. This H_2_O_2_ also increase oxidative stress but to lesser extent. There are various isoforms of SOD such as manganese, zinc and copper, and varies in cellular distribution such as zinc and copper highly available in glial cells, whereas manganese in erythrocytes and neurons [181]. Hesperidine found to increase the activity of superoxide dismutase to reduce oxidants levels in the brain [1,145].

**Figure 51:**
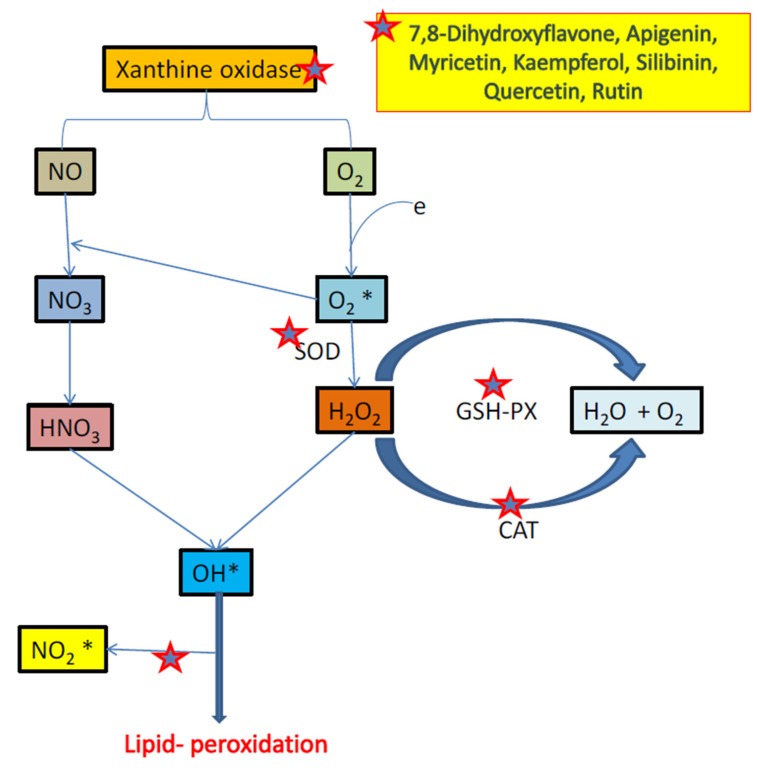
Inhibitory action of flavonoids on Xanthine oxidase pathway**.** NO, nitric oxide; NO_3_, nitrate; HNO_3_, nitric acid; O_2_, oxygen; O_2_*, oxidative free radical; H_2_O_2_, hydrogen peroxide; SOD, superoxide dismutase; GSH-PX, glutathione peroxidase; CAT, catalase; OH*, hydroxyl free radical; NO_2_*, nitrogen dioxide radical.

Xanthine oxidase (XO) pathway has suggested being an important pathway in tissues oxidative injury [182,183,184]. XO and xanthine dehydrogenase are found to catalyze oxidation of hypoxanthine to form xanthine and involve in metabolism process of xanthine to uric acid [182,183,184,185]. Xanthine dehydrogenase is a form of enzyme found to present under normal physiological conditions, but under conditions of ischemic it undergoes configurationally and changes to XO. XO produces oxidative free radicals and also known as pro-oxidant [182]. During reperfusion phase (re-oxidation), XO reacts with oxygen molecule and produces hydrogen peroxide as well superoxide free radical [182,183,184,185,186,187] as shown in Figure 51. In a study, silibin and quercetin inhibited XO activity, thereby reduced oxidative injury [188,189]. According to a study on animal models has proved that luteolin was the most potent XO inhibitor [190]. Several other flavonoids such as 7, 8-dihydroxyflavone, apigenin, myricetin, silibinin, quercetin, rutin and kaempferol tend to exhibit antidepressant action via inhibiting XO [1,145,189]. Rutin is also a powerful free radical scavenger, which finds to inhibit xanthine oxidase enzyme [189].

Cyclo-oxygenase (COX-2) -2 is a rate-limiting enzyme responsible for synthesis of prostaglandin E2, and significantly increases chronic stress-induced depression in rats [191]. Inhibition of COX-2 by celecoxib protects neuron from neuronal injury by suppressing oxidative stress and, thus, mediating its antidepressant effects [192,193,194]. In depression models of rats, COX-2 was found to highly express in hippocampus dentate gyrus and its action is responsible for depression-like behavior. In dentate gyrus, neuroprotective activity was seen by inhibition of COX-2 [192,193,194,195]. Other, critical risk factors such as neuronal apoptosis, neuroinflammatory response and oxidative stress in dentate gyrus, which are responsible for pathophysiology of neuronal damage and depression, were found to be suppressed after inhibition of COX-2. N-acetylcysteine significantly reduces dendritic spine defects and level of oxidative stress, resulted from over-expression of COX-2 and also significantly reduces depressive behavior in rats [191,192,193,194,195]. Therefore, the targeted inhibition of enzyme COX-2 seems to enhance protection against neurodegeneration and oxidative stress in depression as shown above in Figure 48.

### 3.4. Flavonoids and BDNF Expression

Brain derived neuronal factor is a brain-derived neurotrophic factor, found to be highly abundant in human brain and observed in both blood plasma and serum [196]. BDNF is necessary for morphological protection of neuronal dendrites and axon, maintenance of synaptic plasticity; regulation of neuronal survival and intracellular signaling processes [197]. It has been found to be involved in several neurological and psychological disorders such as depression, anxiety, schizophrenia, Alzheimer’s disease, eating disorders, addiction and epilepsy, etc. [198,199]. Hippocampal BDNF expression is regulated by physical exercise, medication, exercise, social interaction and sensory input [200]. It enhances neuronal activity of brain, and this respective positive feedback-loop preserves the state of mind to be active [200]. In contrast, reduction in activity dependent expression of BDNF and neuronal activity is consequence of disruption of BDNF expression caused by stress, epigenetic processes [201]. In postpartum of a brain sample of a depressed patient, reduced level of hippocampal BDNF was seen, which cause atrophy of the hippocampus and also makes it a prominent biomarker in depression etiology. Recently, in several clinical studies, reduction in BDNF levels of plasma and serum was also observed in MDD patient [201,202,203].

In recent studies, extensive investigations have suggested the involvement of BDNF in nervous system is regulated by binding mainly to TrkB [204,205,206]. Precipitating stress factors, i.e., chronic stress can cause a decrease in BDNF support thereby reduces antiapoptotic control of BCL-2 and decreasing the survival of neurogenic cells. This may have negative consequences for hippocampal function and eventually lead to development of depression [204,205,206]. In addition, BDNF activates intracellular tyrosine-kinase activity by binding to TrkB, leading to autophosphorylation of TrkB, phospholipase C-gamma pathway, mitogen-activated-protein kinase pathway, phosphatidyl-inosine 3-kinase pathway and also of other signaling pathways [204,205]. Finally, the CREB response element is activated at the Ser133 site of binding protein (CREB), which results in increase in expression of BCL-2 and BDNF genes and enhances neurogenesis, synaptic plasticity and promotes neuronal survival [204,205,206,207,208]. Besides, development functions and survival of neurons, BDNF-TrkB also increases development of dendrite spine, providing structural basis for formation of synapse, which improves transduction synapse efficiency [204,205,206,207,208,209].

In various animal stress models such as foot shocks, immobilization stress, early maternal deprivation and social defeat, a significantly reduction in hippocampal BDNF level and expression was seen, especially in dentate gyrus [209,210]. Moreover, exogenous corticosterone tends to reduce in hippocampal BDNF expression, reflecting towards involvement hippocampal glucocorticoids in regulating BDNF expression [211]. Hence, stress stimulated the hypothalamic-pituitary-adrenal axis, and then increases glucocorticoids, which ultimately leads to reduce activity of BDNF [204,205,206,207,208,209,210]. In addition, decreased BDNF levels in knockouts or precursors of the BDNF gene may impede the behavioral antidepressants effect [205,206,207,208]. In recent years, there are enough studies to prove the key role of antidepressant treatment in curing depression through regulation of brain BDNF and via activation of TrkB receptors [212,213].

**Figure 52:**
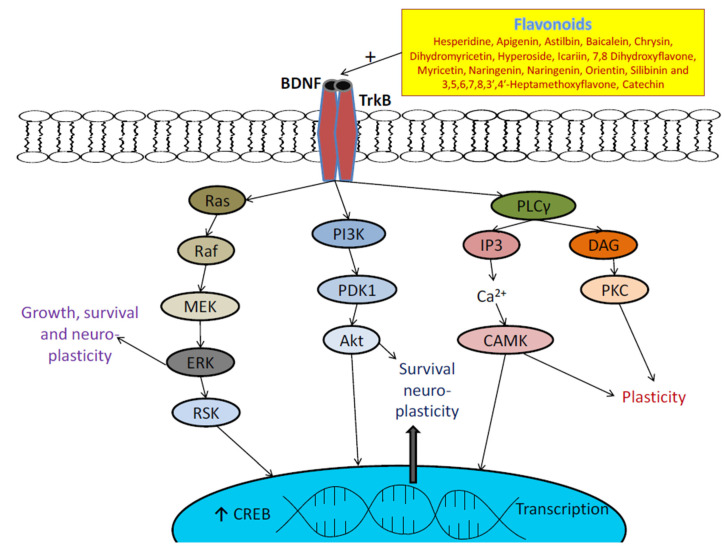
Effect of flavonoids on BDNF/TrkB signalling pathway**.** BDNF, brain-derived neurotrophic factor; TrkB, tropomyosin receptor kinase B; Ras, rapidly accelerated sarcoma; Raf, rapidly accelerated fibrosarcoma; MEK, mitogen-activated protein kinase; ERK, extracellular regulated kinase; RSK, ribosomal S6 kinase; P13K, phosphoinositide 3-kinases; PDK1, 3-phosphoinositide-dependent kinase 1; Akt(PKB), protein kinase B; PLCγ, phospholipase C gamma; IP3, inositol trisphosphate; CAMK, Ca2+/calmodulin-dependent protein kinase; DAG, diacylglycerol; PKC, protein kinase C; CREB, cAMP-response element binding protein.

Various isolated flavonoids have found to reversed reduced level of BDNF and shown anti-depressant action by increasing BDNF expression [1,30,145]. These flavonoids include hesperidine, apigenin, astilbin, baicalein, chrysin, dihydromyricetin, hyperoside, icariin, 7,8 dihydroxyflavone, myricetin, naringenin, naringenin, orientin, silibinin and 3,5,6,7,8,3′,4′-heptamethoxyflavone, etc. [1,41,49,54,55,60,61,68,80,87,92,94,104,122,128,136,137]. In pre-clinical studies, these flavonoids increased hippocampal BDNF level, modulate neuronal network, maintain neuronal plasticity and regulate neurogenesis, neuronal maturation and synaptogenesis in rodent brain [214,215,216]. Unlike synthetic antidepressant, these flavonoids found to prevent both stress-induced and corticosterone-mediated decrease in BDNF expression [214,215,216] as shown in Figure 52. Therefore, BDNF/TrkB signalling found to have a major and significant impact on antidepressant effects of flavonoids [214].

In Table 5, isolated flavonoids have been summarized with their respective dose, route of administration and mechanism of action.

## 4. Future Aspects

In the present literature, flavonoids found to receive much more attention over past fifteen years and number of beneficial actions has been described. The interaction of flavonoids with mental health appears to be very significant. The present reviewed literatures are promising to suggest that flavonoids could be a useful food compound. On other side, as most of research includes only in-vitro studies, which makes difficult to conclude about usefulness of flavonoids as diet.

Nowadays, the main benefit of nutraceuticals as compared to allopathic treatment depends on the patient’s compliance, and which seem to have increased. Additionally, they may be synergistically combined with standard medical drugs, which are currently being in use for depression, such as SSRIs or MAO inhibiters, etc. However, at present, there are insufficient clinical reports to vouch for efficacy of nutraceuticals for treatment of depression and current information is lies on mainly pre-clinical experimental studies. This scarcity of documented clinical data suggest the future need of clinical trials. During these studies, there should be considerable attention on specific populations with stress; either it is arising due to environmental factors (e.g., stress due to work, daily life or changes in social life) or genetic factors.

**Figure 53:**
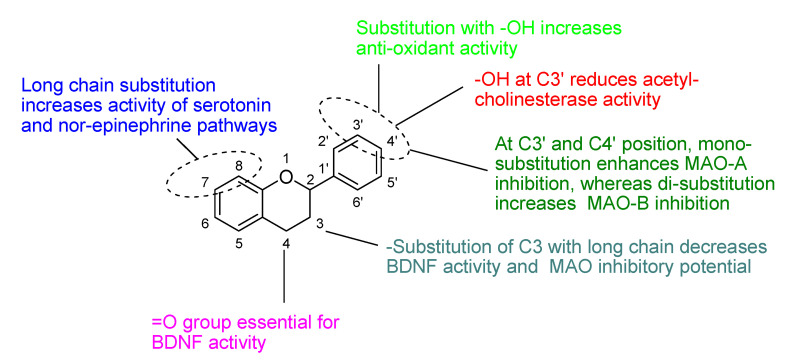
SAR of Flavonoids.

In the present review, a specific emphasis is given on structure base activity relationship of different reported flavonoids, which would help in screening of potent molecules having ability to undergo clinical trials. On the basis of above discussed SAR of flavonoids some remarkable points have been concluded as shown in Figure 53. These remarkable outcomes would help in designing future plant based new phyto-medicine. Furthermore, the impact of flavonoids treatment on patients’ metabolism and lipidomics profiles could help in evaluation whether these flavonoids are targeting to particular pathways involved in the pathogenesis of depression.

Although there are positive results about antidepressant-like action of certain flavonoids at pre-clinical level, hence the side effects of prolonged use should be investigated, including toxicity study and possible pharmacological interactions, determination of safety, bioavailability and tolerability in human body. These studies may eventually prove that certain flavonoids are safer options in clinical practice for treatment of depression. Furthermore, there are not sufficient methods available for measuring in-vivo oxidative damage and even, objective endpoints measurement is also remains difficult. Hence, there is also requirement to improve analytical methods to allow for more data collection on excretion and absorption of flavonoids. In conclusion, present in-vivo studies give a picture of hope for the future extensive studies.

## 5. Conclusions

Flavonoids are considered to be very interesting bioactive polyphenols which obtained from various plant and affecting adult brain. In the present study, we tried to compile and analyzed the effects of several flavonoids in depression and their effects on behavioural, molecular and physiological level in rodents. Interestingly, we have observed that one molecule can exhibit anti-depressive action acting via different mechanisms including increase in neurogenesis, neurotransmitters, BDNF level, modulation of receptors and antioxidant action, whereas, clinical data are still limited; sophisticated future research is therefore needed to confirm the results of the current experimental studies, to completely understand the mechanisms of action with bio-transformations of their metabolism in human body and interactions with depression-related receptors.

## Figures and Tables

**Table 1:** Flavones and their Structure activity relationship.

Sr. No.	Flavones	C_5_	C_6_	C_7_	C_8_	C_3′_	C_4′_
1.	7,8-Dihydroxyflavone			-OH	-OH		
2.	Amentoflavone	-OH		-OH	-C_15_H_12_O_5_		-OH
3.	Apigenin	-OH		-OH			-OH
4.	Baicalein	-OH	-OH	-OH			
5.	Chrysin	-OH		-OH			
6.	Luteolin	-OH		-OH		-OH	-OH
7.	Nobiletin	-OCH_3_	-OCH_3_	-OCH_3_	-OCH_3_		-OCH_3_
8.	Orientin	-OH		-OH	-C_6_H_11_O_5_	-OH	-OH
9.	Vitexin	-OH		-OH	-C_6_H_11_O_5_		-OH

**Table 2:** Flavonols and their structure activity relationship.

Sr. No.	Flavonols	R	C_5_	C_6_	C_7_	C_8_	C_3′_	C_4′_	C_5′_
1.	3,5,6,7,8,3′,4′-Heptamethoxyflavone	-CH_3_	-OCH_3_	-OCH_3_	-OCH_3_	-OCH_3_	-OCH_3_	-OCH_3_	
2.	Fisetin	H			OH		OH	OH	
3.	Hyperoside	(C_6_H_11_O_5_)	-OH		-OH		-OH	-OH	
4.	Icariin	H	OH		-O(C_6_H_11_O_5_)	C_5_H_9_		OCH_3_	
5.	Isoquercitrin	-(C_6_H_11_O_5_)	OH		OH		OH	OH	
6.	Kaempferitrin	(C_6_H_11_O_4_)	-OH		-O(C_6_H_11_O_4_)			-OH	
7.	Kaempferol	H	OH		OH			OH	
8.	Kaempferol-3-O-β-D-glucose	-(C_6_H_11_O_5_)	OH		OH			OH	
9.	Miquelianin	-(C_6_H_9_O_6_)	OH		OH		OH	OH	
10.	Myricetin	H	OH		OH		OH	OH	OH
11.	Myricitrin	-(C_6_H_11_O_4_)	OH		OH		OH	OH	OH
12.	Quercetin	H	OH		OH		OH	OH	
13.	Quercetin-3-O-β-D-glucose	-(C_6_H_11_O_5_)	OH		OH		OH	OH	
14.	Quercitrin	-(C_6_H_11_O_4_)	OH		OH		OH	OH	
15.	Rutin	-(C_12_H_21_O_9_)	OH		OH		OH	OH	

**Table 3:** Flavanones and their structure activity relationship.

Sr. No.	Flavanone	C_5_	C_7_	C_3′_	C_4′_
1.	Hesperidin	-OH	-O(C_12_H_21_O_9_)	-OH	-OCH_3_
2.	Isosakuranetin-5-*O*-rutinoside	-O(C_12_H_21_O_9_)	-OH		-OCH_3_
3.	Liquiritin		-OH		-O(C_6_H_11_O_5)_
4.	Naringenin	-OH	-OH		-OH
5.	Naringin	-OH	-O(C_12_H_20_O_9_)		

**Table 4:** Flavanonols and their Structure activity relationship.

Sr. No.	Flavanonols	R’	C_5_	C_6_	C_3′_	C_4′_	C_5′_
1.	Astilbin	-O(C_6_H_11_O_4_)	-OH	-OH	-OH	-OH	
2.	Dihydromyricetin	-H	-OH	-OH	-OH	-OH	-OH

**Table 5:** Isolated flavonoids with anti-depressant action.

Flavones						
Isolated Bioactive Flavonoids	Doses	Route	Animal Species	Treatment Duration	Mechanism of Actions	References
7,8-Dihydroxyflavone	1, 3, and 10 mg/kg	Intra-gastric	Male Swiss mice	60 min before test	Modulation of nitric oxide signalling pathway Increased BDNF level in prefrontal cortex and hippocampus	[41]
	10 and 20 mg/kg	Intraperitoneal	Male C57BL/6 mice	28 days	Permeable to the BBB and mimics hippocampal brain-derived neurotrophic factor action Acted as TrkB receptor-specific agonist	[42]
	5 mg/kg	Oral	Male C57BL/6 mice	21 days	Permeable to the BBB and mimics hippocampal brain-derived neurotrophic factor action Acted as TrkB receptor-specific agonist	[43]
Amentoflavone	6.25, 12.5, 25, or 50 mg/kg	Oral	Male Swiss albino mice	3 days	Interacted with serotonergic (especially, 5- HT_2_ receptors) and noradrenergic (especially, *α*_1_ and *α*_2_ receptors)	[45]
Apigenin	12.5 and 25 mg/kg	I.p.	Male ddY mice	1 h	Regulated dopaminergic system	[47]
	20 mg/kg	I.g.	Male Sprague–Dawley rats	21 days	Inhibited IL-1 production Inhibited NLRP3 inflammasome expression Up-regulated PPAR expression	[48]
	20 and 40mg/kg	Oral	Male ICR mice	21 days	Up-regulated BDNF concentrations in the hippocampus	[49]
	25, 50 mg/kg	I.p.	Male ICR mice	7 days	Inhibited nitric oxide synthase Inhibited cyclooxygenase-2	[51]
Baicalein	1, 2, or 4 mg/kg	I.p.	Male Kunming mice	7 and 21 days	Reversed in the reduction of extracellular ERKs phosphorylation Enhanced level of hippocampal BDNF expression	[54]
	10, 20, and 40 mg/kg	I.p.	Male Sprague–Dawley rats	14 days	Prevented decrease of BDNF level and dopamine concentrations in hippocampus	[55]
	10, 20, or 40 mg/kg	Oral	Male Wistar rats	35 days	Decreased COX-2 activity and as well its expression Reduced of PGE2 levels in brain	[56]
Chrysin	5 and 20 mg/kg	Oral	Female C57B/6J mice	28 days	Increased BDNF and nerve growth factor levels in cortex prefrontal and hippocampus Antioxidant activity	[60]
	5 and 20 mg/kg	Oral	Male C57B/6J mice	14 days	Increased brain-derived neurotrophic factor synthesis Increased serotonin level in hippocampus	[61]
	5 and 20 mg/kg	Oral	Female C57B/6J mice	28 days	Reverse the decreases hippocampal 5-HT levels Reduction in IL-1β, TNF-α, IL-6 and kynurenine levels Increased the caspases activities in cerebral	[62]
Luteolin	50 mg/kg	Oral	Male ICR mice	23 days	Suppressed hippocampal endoplasmic reticulum stress via inhibiting the expression of endoplasmic reticulum stress related proteins	[63]
	5 or 10 mg/kg	Oral	Male ICR mice	30 min before test	Potentiated the GABA_A_ receptor- Cl^−^ ion channel complex	[64]
Nobiletin	20, 50, or 100 mg/kg	Oral	Male ICR mice	After 60 min	Activated serotonergic, noradrenergic and dopaminergic systems	[66]
Orientin	20 and 40 mg/kg	Oral	Male Kunming mice	21 days	Increased BDNF level Increased in levels of serotonin, and nor-epinephrine levels in the hippocampus and prefrontal cortex Improved central oxidative stress, neuroplasticity and neurotransmission MAO inhibition	[68]
Vitexin	10,20 and 30 mg/kg	Oral	Male BALB/c mice	60 min before test	Increased monoamines level in synaptic cleft Interacted and modulated noradrenergic α_2_, dopaminergic D_1_, D_2_, D_3_ and serotonergic 5-HT_1A_ receptors	[77]
**Flavonols**						
3,5,6,7,8,3′,4′-Heptamethoxyflavone	50 mg/kg	S.c.	C57BL/6 mice	25 days	Increased in hippocampal BDNF concentration Increased in neurogenesis and neuroplasticity in the hippocampus	[80]
Fisetin	10 or 20 mg/kg	Oral	Male ICR mice	4 days	Inhibited MAO activity Up-regulation of serotonin in and nor-epinephrine levels	[83]
	20, 40, or 80 mg/kg	Oral	Male ICR mice	7 days	Antagonized nitrite levels and iNOS mRNA expression via modulating NF-*κ*B Reversed LPS-induced overexpression of proinflammatory cytokine (especially, IL-6, IL-1β and TNF-α)	[84]
	5 mg/kg	Oral	Male ICR mice	21 days	Increased phosphorylation and activation of TrkB (pTrkB) in the hippocampus	[85]
Hyperoside	2.5, 5, and 10 μg/mL	I.p.	PC12 cell line	4 h	Cytoprotective action via increased in expression of BDNF and as well CREB by activating signalling pathway, i.e., AC–cAMP–CREB	[87]
	10, 20, or 40 mg/kg	I.p.	Male CF1 mice	14 days	Activated dopaminergic system via D_2_-DA receptors	[88]
	0.6 mg/kg	Oral	Male CD rats	14–56 days	Modulated hypothalamic-pituitary-adrenal axis by reduction of plasma ACTH and corticosterone concentration	[89]
Icariin	20 and 40 mg/kg	Oral	Male Sprague–Dawley rats	35days	Anti-oxidant action Inhibited activation of NF-κB signaling and also, NLRP3 -inflammasome/caspase-1/IL-1b axis Anti-inflammatory action	[91]
	5 and 10 mg/kg	Oral	Male C57BL/6J mice	28 days	Increased BDNF expression Inhibited the increases in serum TNF-α and IL-6 level Restored the impairment of gluco-corticoid sensitivity	[92]
	20 and 40 mg/kg	Oral	Male Sprague–Dawley rats	35 days	Restored the negative feedback regulation of the HPA- axis Decreased the expression levels of FKBP5 and SGK1	[93]
	60 mg/kg	Oral	Male Sprague–Dawley rats	21 days	Increased in hippocampal BDNF concentrations Reversed CORT-induced depression via regulating disturbed metabolic pathways	[94]
Isoquercitrin	0.6 mg/kg	Oral	Male CD rats	14–56 days	Modulated hypothalamic-pituitary-adrenal axis by reducing plasma corticosterone and ACTH concentration	[89]
	2.5 mg/kg	Oral	Male Sprague–Dawley rats	14 days	Protected from oxidative stress	[95]
Kaempferitrin	1, 5, 10, or 20 mg/kg	Oral	Male Swiss Webster mice	4 days	Interacted with presynaptic 5-HT1A receptors and	[98]
Kaempferol	30 mg/kg/day	Oral	Male ICR mice	14 days	Increased expression of plasma β-endorphin levels or hypothalamic POMC mRNA	[100]
	0.35 mM/kg	I.p.	Male Swiss mice	60 min prior to the test	Increased in NE, DA and 5-HT and also reduced 5-HT metabolism	[101]
Kaempferol-3-O-β-D-glucose	0.35 mM/kg	I.p.	Male Swiss mice	60 min prior to the test	Increased in NE, DA and 5-HT and also reduced 5-HT metabolism	[101]
Miquelianin	0.6 mg/kg	Oral	Male CD rats	14 days	Modulated hypothalamic-pituitary-adrenal axis by reducing plasma ACTH and corticosterone concentration	[89]
Myricetin	50 mg/kg	I.p.	Male C57BL/6 mice	21 days	Increased BDNF concentrations in hippocampus Reduced oxidative stress	[104]
Myricitrin	10 mg/kg	I.p.	Male Balb/C mice	21 days	Facilitated hippocampal neurogenesis	[106]
Quercetin	2.5, 5, 10, 20 and 40 mg/kg	Oral	Male Sprague–Dawley rats	14 days	Protected from oxidative stress	[95]
	0.35 mM/kg	I.p.	Male Swiss mice	60 min prior to the test	Increased in NE, DA and 5-HT and also reduced 5-HT metabolism	[101]
	50 or 100 mg/kg	I.p.	Male Wistar rats	21 days	Attenuated depressive-like behaviours	[108]
	40 and 80 mg/Kg	Oral	Male olfactory bulbectomy rats	14 days	Neuroprotective effects via microglial inhibitory pathway Suppressed oxidative-nitrosative stress mediated neuroinflammation-apoptotic cascade	[109]
	25 mg/kg	Oral	Female Swiss mice	14 days	Antagonised NMDA receptors Inhibited synthesis of nitric oxide	[211]
Quercetin- 3-O-β-D-glucose	0.35 mM/kg	I.p.	Male Swiss mice	60 min prior to the test	Increased in NE, DA and 5-HT and also reduced 5-HT metabolism	[101]
Quercitrin	30 mg/kg/day	Oral	Male ICR mice	14 days	Increased expression of plasma β-endorphin levels and hypothalamic POMC mRNA	[100]
Rutin	0.3, 1, 3, 10 mg/kg	Oral	Male Swiss mice	4 days	Increased the concentration of serotonin and noradrenaline in the synaptic cleft	[8]
	5 and 10 mg/kg	Oral	Male Sprague–Dawley rats	14 days	Protected from oxidative stress	[95]
**Flavanones**						
Hesperidin	25, 50, or 100 mg/kg	Oral	Male albino Wistar rats	21 days	Attenuated hyperglycaemia and restored brain monoamines level Increased the neurogenesis and brain-derived neurotrophic factor levels	[117]
	0.01, 0.1, 0.3, and 1 mg/kg	I.p	Male Swiss mice	21 days	Interacted with the *κ*-opioid receptor	[118]
	0.01, 0.1, 0.3, and 1 mg/kg	I.p.	Male Swiss mice	21 days	Interacted with the 5-HT_1A_ receptors Antioxidant effect	[119]
	25 and 50 mg/kg	Oral	Male ICR mice	21 days	Increased ERK phosphorylation and BDNF expression in hippocampus	[121]
	0.01, 0.1, 0.3, and 1 mg/kg	I.p.	Male Swiss mice	21 days	Increased hippocampal brain-derived neurotrophic factor levels Inhibited l-arginine-NO-cGMP pathway Decreased hippocampal nitrate/nitrite (NOX) levels	[122]
	0.4, 4, 8, 16, and 32 mg/kg	Oral	Male imprinting control region mice	After 1 h	Increased neuronal level of the 5-HT and dopamine	[119]
	50 mg/kg	Oral	Male C57BL/6 mice	13 days	Increased in hippocampal BDNF and nerve growth factor concentrations Modulation of pro-inflammatory cytokine Maintained brain plasticity Inhibition of acetylcholinesterase activity	[217]
Isosakuranetin-5-*O*-rutinoside	15 and 30 mg/kg	Oral	Male ICR mice	24, 18, and 1 h before test	Significantly inhibited depression-like behaviours	[123]
Liquiritin	10, 20 and 40 mg/kg	G.i.	Male mice	30 min before Sample	Increased in 5-HT and NE levels in hippocampus, hypothalamus and cortex	[124]
Isoliquiritin	10, 20 and 40 mg/kg	G.i.	Male mice	30 min before sample	Increased 5-HT and NE levels in hippocampus, hypothalamus and cortex	[124]
Naringenin	5, 10 and 20 mg/kg	Oral	Male ICR mice	14 days	Increased serotonin, dopamine, norepinephrine and glucocorticoid receptor levels in the brain hippocampus	[130]
	20 mg/kg	Oral	Male ICR mice	21 days	Activation of hippocampal BDNF signalling pathway	[128]
Naringin	50 and 100 mg/kg	I.p.	Male Wistar rats	14 days	Significantly inhibited DOX-induced raise in plasma corticosterone, TNF-α and IL-1β levels in hippocampus Modulated of 5-HT_1A_ and kappa-opioid receptors	[133]
**Flavanonols**						
Astilbin	10, 20, or 40 mg/kg	I.p.	Male C57BL/6J mice	21 days	Upregulated mono-aminergic neurotransmitters Activated of BDNF signalling pathway Inhibition of MAO activity	[136]
Dihydromyricetin	10 and 20 mg/kg	I.p.	Male C57BL/6J mice	7 days	Increased mRNA expression for BDNF in the hippocampus Inhibited neuro-inflammation	[137]
**Flavanols**						
Catechin	88.6 and 58.9 µM	-	Wistar male rat	-	Inhibition of MAO activity	[139]
Epicatechin	88.6 and 58.9 µM	-	Wistar male rat	-	Inhibition of MAO activity	[139]
Epigallocatechin gallate	500 ng/mL	-	Sprague–Dawley rat brains	24 h	Reduced neuroinflammation	[141]
**Other flavonoids**						
Silibinin	100 and 200 mg/kg	Oral	Either sex Wistar rats	14 days	Altered immunological, endocrine and monoamines systems such as 5-HT, DA, NE, MDA formation, TNF-α, IL-6 and BDNF levels in hippocampus and cerebral cortex.	[143,144]

## Data Availability

The data that support our specific findings in this review are available from the authors upon reasonable request.

## Abbreviations

5-HT	5-Hydroxy tryptophan
MDD	Major depression disorder
MAO	Monoamino oxidase
D1	Dopamine 1
D2	Dopamine 2
COX	Cyclooxygenase
XO	Xanthine oxidase
NOS	Nitric oxide synthase
LOX	Lipooxygenase
IP_3_	Inositol triphosphate
PKC	Phosphokinase C
BDNF	Brain derived neurotrophic factor
CREB	cAMP-response element binding protein
NO	Nitric oxidase
SOD	Superoxide dismutase
BCL-2	B-cell lymphoma 2
TrkB	Tropomyosin receptor kinase B
